# Construction of Adverse Outcome Pathway (AOP) for Triphenyl Phosphate (TPHP)–Induced Autism Spectrum Disorder (ASD) and Risk Identification of Neurodevelopmental Toxicity of Aryl Organophosphorus Flame Retardants (OPFRs) Based on Multidimensional Bioinformatic Data

**DOI:** 10.3390/ijms27198769

**Published:** 2026-09-30

**Authors:** Yonghang Li, Kaidong Wang, Ai Qi, Qi Liu, Ge Shi, Min Huang

**Affiliations:** 1 School of Public Health, Ningxia Medical University, No. 1160, Shengli Street, Xingqing District, Yinchuan 750001, China; lyh0608lucky@163.com (Y.L.);; 2 Key Laboratory of Environmental Factors and Chronic Disease Control, Ningxia Medical University, No. 1160, Shengli Street, Xingqing District, Yinchuan 750001, China

**Keywords:** triphenyl phosphate, autism spectrum disorder, transcriptomics, simulated gene knockout, adverse outcome pathway

## Abstract

Triphenyl phosphate (TPHP) is a widely used plasticizer and organophosphate flame retardant extensively applied in various industrial and consumer products. Due to its pervasive environmental exposure and detection in human samples such as amniotic fluid and breast milk, TPHP has become a pollutant of significant concern in recent years for its potential neurodevelopmental impacts on humans. This study employed a multimethod approach integrating network toxicology, transcriptomics, in silico gene knockout, molecular docking, and molecular dynamic simulations to investigate TPHP’s potential toxic role in autism spectrum disorder (ASD). An adverse outcome pathway (AOP) was constructed, revealing that TPHP exposure may induce and exacerbate ASD by downregulating *GAD1*—the core gene encoding the GABA synthesis rate–limiting enzyme (glutamate decarboxylase)—leading to an excitation–inhibition imbalance in the brain. The batch molecular docking of 85 organophosphate flame retardants (OPFRs) (widely detected in human and biological matrices) with the GAD1 protein identified aryl–OPFRs as disruptors targeting *GAD1* to induce neurodevelopmental toxicity. In summary, this research provides mechanistic insights into how TPHP promotes ASD progression and offers a valuable chemical reference for assessing the neurodevelopmental toxicity of OPFRs.

## 1. Introduction

Traditional brominated flame retardants (BFRs) are gradually being banned and restricted worldwide. To meet the current demand for flame retardants, organophosphorus flame retardants (OPFRs) have become their main alternatives and are widely used, and triphenyl phosphate (TPHP) is a widely used plasticizer and organophosphorus flame retardant, which is widely used in different industries and consumer products [1]. Since TPHP is added to materials by physical means rather than chemical bonding, TPHP can easily penetrate into the environment through volatilization and mechanical abrasion [2]. In recent years, the use of TPHP has increased significantly worldwide, resulting in its distribution in a variety of environmental media, such as air [3], soil [4], dust [5], and water and sediment [6]. Importantly, TPHP and its metabolites have also been widely detected in human samples, such as hair, placenta, blood, and urine samples. Therefore, there is a wide range of types of environmental and population exposures to TPHP, TPHP poses an environmental health risk, and the potential health effects of TPHP have attracted widespread attention. In recent years, multiple studies have revealed that TPHP exerts various toxic effects, including endocrine−disrupting toxicity [7], hepatotoxicity [8], neurodevelopmental toxicity [9], reproductive developmental toxicity [10], and immunotoxicity [11]. It is noteworthy that early life developmental toxicity [12] is a major concern for human beings. Notably, the developing brain early in life is particularly sensitive to exogenous toxins, and exposure to very low levels of the chemical may result in persistent neurobehavioral damage [13]. Epidemiological and toxicological studies have demonstrated the developmental neurotoxicity of TPHP exposure. A study exploring the developmental neurotoxicity of TPHP in fish using zebrafish larvae as a model showed that exposure to environmentally relevant concentrations of TPHP affected the development of the central nervous system, thereby promoting developmental neurotoxicity in early zebrafish larvae [14]. A study exploring the developmental neurotoxicity of TPHP using rats as a model showed that exposure to the flame retardant TPHP during pregnancy and the early postnatal period resulted in a significant increase in the risk for the developmental neurotoxicity of the flame retardant TPHP in the early postnatal period. The flame retardant TPHP causes long−term neurobehavioral and neurochemical dysfunction [15]. The results from a population−based study of OPFR exposure in pregnant women and children and its potential neurodevelopmental outcomes, which measured metabolites of OPFRs in the urine of pregnant women in the CHAMACOS birth cohort (*n* = 310), showed that TPHP metabolites were detected at a rate of up to 79%, suggesting that as the maternal prenatal levels of TPHP metabolites increased, they led to increased levels of whole–measurement IQ and working memory levels in postnatal children [16]. These studies suggest that TPHP exposure exhibits developmental neurotoxicity.

Autism spectrum disorder (ASD) is a highly heterogeneous neurodevelopmental disorder characterized by impairments in communication and social behavior, divided into two main types, idiopathic and secondary, with idiopathic ASD being the more common, often thought to involve multiple genes and a complex interaction with environmental factors such as exogenous chemical exposure, maternal infection during pregnancy and drug exposure [17]. Complex interactions are closely associated with the development of idiopathic ASD. Numerous epidemiological studies have shown that environmental factors may increase the risk of ASD, which may contribute to up to 50% of the variation in risk of ASD [18], and several studies have found that exogenous chemicals in the environment exacerbate the risk of ASD [19,20,21]. The frontal cortex is a key component of the “social brain,” a major brain region that is involved in social cognition and influences social behavior. Converging evidence across studies suggests that abnormalities in the frontal cortex are an important feature of ASD [22,23,24,25], and exploring the potential neuropathological mechanisms of the frontal cortex in ASD patients is an important direction for future research on ASD. Given that 40–50% of the etiology of ASD is attributed to environmental factors [26], the present study focuses on TPHP and ASD, aiming to investigate the potential molecular mechanisms of the toxic effects of TPHP exposure in frontal cortex tissues that induces the onset and development of ASD.

Traditional animal models for exploring the molecular mechanisms of the toxic effects of exogenous chemicals on disease onset and progression, and for assessing their chemical toxicity, are costly and time–consuming [27,28]. The rapid growth in the number of chemicals has made it impractical to assess the chemical toxicity of tens of thousands of new chemicals using animal models in multiple fields such as healthcare, industry, and agriculture [29]. Computational toxicology based on the big data of chemical toxicity is a promising alternative approach to predicting the toxic potential of chemicals and exploring the toxic effects of chemicals in inducing disease development and their potential mechanisms of toxicity [30,31]. The present study integrates multiple computational toxicology methods to efficiently investigate the toxicity of TPHP and its potential molecular mechanisms in the development of ASD and to elucidate the inherent complex toxicity mechanisms and identify potential targets for intervention in a time−saving and efficient manner through toxicity analysis and histological techniques.

Based on the comprehensive analysis of absorption, distribution, metabolism, excretion, and toxicity (ADMET)–related information about TPHP using the ADMETlab 3.0 database, this study integrated network toxicology, transcriptomics, and animal experimental analysis. Employing multiple technical approaches including machine learning, simulated gene knockout, molecular docking, and molecular dynamic simulation, an adverse outcome pathway (AOP) for the TPHP exposure–induced occurrence and development of autism spectrum disorder (ASD) was constructed, with key intervention targets serving as the molecular initiating event (MIE). Further, using a batch molecular docking approach, we evaluated the binding affinities of 85 types of OPFRs—widely detected in human and biological matrices—with the key intervention targets [32]. This allowed us to determine the influence of substituent types of OPFRs on the binding affinity to these key targets, thereby identifying which OPFRs are likely to cause an imbalance between brain excitatory and inhibitory signaling via these targets and subsequently induce neurodevelopmental toxicity. Our findings provide a theoretical foundation for understanding the health risks associated with OPFRs and for formulating strategies to mitigate their impact on ASD. Environmental interventions targeting modifiable risk factors may offer promising avenues for the prevention and treatment of ASD.

## 2. Results

### 2.1. Evaluating ADMET Properties of TPHP Based on ADMETlab 3.0 Database

TPHP primarily enters the human body through the respiratory tract and gastrointestinal tract, undergoing processes of absorption, distribution, metabolism, and excretion, leading to its accumulation in various organs. We evaluated the ADMET properties of TPHP based on the ADMETlab 3.0 database. The permeability of TPHP in Madin–Darby Canine Kidney (MDCK) cells was higher than the optimal permeability rate of 7.08 × 10^−6^ cm/s, and its permeability in Human Colorectal Adenocarcinoma Cells (Caco–2) exceeded the moderate permeability standard of 2 × 10^−6^ cm/s, indicating that TPHP possesses strong intestinal permeability (Table S1). The plasma protein binding (PPB) rate of TPHP is approximately 98.897%, exceeding the high standard of 90% for PPB. The fraction unbound in plasma (Fu) is approximately 0.432%, which is below the low standard of 5% for the plasma free fraction. This indicates that TPHP can persist in the bloodstream (Table S1). The probability of TPHP serving as a substrate for CYP1A2, CYP2C19, CYP2C9, CYP2D6, CYP3A4, and CYP2B6 is not zero, indicating that TPHP possesses certain anti–metabolic properties (Table S1). The clearance rate (CL) of TPHP falls within the moderate clearance range of 5–15 mL/min/kg but is close to the low clearance standard of 5 mL/min/kg, indicating that TPHP possesses certain anti–excretion properties (Table S1). Therefore, TPHP exhibits characteristics such as a high absorption rate, anti–metabolism, and anti–excretion, which lead to its prolonged presence in the human body and may cause persistent damage to human organs. Notably, TPHP can cross the blood–brain barrier (BBB), indicating that TPHP is a neurotoxin.

### 2.2. Acquisition of Intersection Targets Between TPHP and ASD

Network toxicology is employed to characterize the toxicological properties of chemicals, elucidate their underlying mechanisms, and predict major toxic components and targets. It represents an advanced methodology for predicting the mechanisms of action and targets of environmental pollutants. We utilized network toxicology to explore the potential pathogenic role of TPHP in ASD. A total of 9061 targets related to TPHP were collected from the CTD and SEA databases. Through the GeneCards and OMIM databases, 3925 targets associated with ASD were screened. Ultimately, 1784 intersection targets related to ASD induced by TPHP exposure were identified (Figure 1A).

### 2.3. Analysis and Construction of Protein–Protein Interaction Network for TPHP–Induced ASD Potential Targets

To further identify the potential key targets of TPHP exposure–induced ASD, we performed a protein–protein interaction (PPI) network analysis on the aforementioned intersection targets using the STRING database. The analysis was conducted with the species set to “*Homo sapiens*,” a high confidence threshold of 0.900, and the exclusion of target genes that did not interact with any other proteins. Subsequently, the network analysis tool in Cytoscape 3.8.0 software was utilized to calculate the topological parameters of the analyzed targets from the STRING database. Based on the criteria of a degree value greater than 5, 852 targets were removed from the total of 1371 targets, resulting in the screening of 519 potential core targets of TPHP–induced ASD (Table S2).

### 2.4. Differential Expression Analysis of Frontal Cortex Tissue in GSE28521 Dataset

The GSE28521 dataset, derived from the GPL6883 platform and comprising frontal cortex tissue samples, underwent normalization preprocessing and data correction (Figure 1B), followed by differential expression analysis. By analyzing DEGs between the control and ASD groups in the frontal cortex tissue of the GSE28521 dataset, with the criteria of “|log2FC (Fold Change)| ≥ 0.585 and *p*-value < 0.05” for DEG identification, a total of 69 DEGs were identified in the ASD group compared to the control group. Among these, 48 genes were upregulated, and 21 genes were downregulated (Figure 1C).

### 2.5. Intersection of Targets and Functional Pathway Analysis from Network Toxicology and Transcriptomics

The intersection of network toxicology analysis via GSE28521 dataset DEGs was conducted to obtain five intersection targets (*LYN*, *PTGS2*, *MSN*, *GAD1*, *GAD2*) (Figure 1D), and subsequently, GO and KEGG analysis was performed to elucidate the molecular mechanism of TPHP–induced ASD toxicity. Using an adjusted *p*-value < 0.05 as the threshold, we identified 320 significant GO terms and eight KEGG pathways. The GO terms included 270 biological process (BP) terms, 19 cellular component (CC) terms, and 31 molecular function (MF) terms. The top 10 GO terms from each of the three categories and the eight KEGG signaling pathways were selected for visualization (Figure 1E,F). GO functional enrichment analysis revealed that in the BP category, the targets were primarily enriched in terms such as response to xenobiotic stimulus, neurotransmitter biosynthetic process and glutamate metabolic process—indicating that these targets play important roles in biological stress response, neurotransmitter metabolism, and amino acid metabolism. In the CC category, the targets were mainly enriched in terms like clathrin–sculpted vesicle, inhibitory synapse and presynapse. In the MF category, the targets were predominantly enriched in terms such as carboxy–lyase activity, carbon–carbon lyase activity and glutamate binding. This suggests that the potential targets of TPHP–induced ASD primarily function by catalyzing the cleavage or formation of specific chemical bonds (via lyases, decarboxylases, oxidoreductases, etc.) and by specifically recognizing and binding key metabolic cofactors (such as vitamin B6/PLP), vitamins, neurotransmitters (e.g., glutamate), membrane lipids (e.g., glycosphingolipids), or cell surface receptors. KEGG pathway enrichment analysis showed that the targets were mainly involved in pathways such as Taurine and hypotaurine metabolism, GABAergic synapse and the NF–kappa B signaling pathway. This indicates that the potential targets of TPHP–induced ASD play crucial roles in amino acid metabolism, neurotransmitter metabolism, immune response, and inflammatory reactions and may serve as potential molecular mechanisms underlying TPHP−induced ASD development.

### 2.6. Expression Validation of Core Targets and ROC Curve Analysis of Diagnostic Efficacy

Based on the intersection of network toxicology and transcriptomic analyses using the GSE28521 dataset, five intersecting targets were identified: *LYN*, *PTGS2*, *MSN*, *GAD1*, and *GAD2*. Further analysis revealed that *GAD1* and *GAD2* were the most frequently involved targets in the KEGG enrichment pathways. Using the GEO database, we validated the expression levels of the two core hub targets, *GAD1* and *GAD2*, in the normalized dataset from the GPL6883 platform using frontal cortex tissues from GSE28521. Compared with normal frontal cortex tissues, *GAD1* expression was significantly downregulated in ASD frontal cortex tissues, while *GAD2* expression was upregulated but not statistically significant (Figure 2A). Subsequently, we assessed the diagnostic efficacy of these two core hub targets by plotting ROC curves and calculating AUC values. The AUC values for *GAD1* and *GAD2* were 0.719 and 0.652, respectively (Figure 2B). As a validation of the GSE28521 dataset, we further utilized the normalized dataset from the GPL15207 platform using frontal cortex tissues from GSE113834 in the GEO database to compare the expression levels of the two core hub targets between the control and ASD groups. Notably, both *GAD1* and *GAD2* expression were significantly downregulated in ASD frontal cortex tissues compared to normal frontal cortex tissues (Figure 2C). The diagnostic efficacy of these two core hub targets was then assessed by plotting ROC curves and calculating AUC values. The AUC values for *GAD1* and *GAD2* were 0.767 and 0.744, respectively (Figure 2D). Collectively, the findings from both the GSE28521 and GSE113834 datasets indicate that *GAD1* possesses good diagnostic capability and was therefore included in subsequent analyses.

### 2.7. Transcriptomic Analysis of Forebrain Samples from Embryonic Day 15 Mice Following Transgenerational Exposure to TPHP

Transcriptomic analysis was performed on mouse forebrain samples on embryonic day 15 exposed to TPHP. Principal component analysis (PCA) revealed significant transcriptomic differences between the control and experimental groups (Figure 3A). Based on differential expression analysis, using the criteria of |log2FC| ≥ 0.585 and adjusted *p*-value < 0.05, a total of 444 differentially expressed genes (DEGs) were identified between the experimental and control groups, of which 297 were upregulated, and 147 were downregulated (Figure 3B). Subsequently, GO and KEGG pathway analyses were conducted on the 444 DEGs to elucidate their potential biological functions and molecular pathways. With a threshold of an adjusted *p*-value < 0.05, a total of 888 significant GO terms (including 735 BP terms, 81 CC terms, and 72 MF terms) and 19 KEGG pathways were obtained. The top 10 GO terms from each of the three categories and all KEGG signaling pathways were selected for visualization (Figure 3C,D). In the BP category, the targets were mainly enriched in terms such as synapse organization and regulation of synaptic plasticity, suggesting their potential roles in synapse–related neural structure formation and cognitive functions. In the CC category, the targets were predominantly enriched in terms including the synaptic membrane, neuron–to–neuron synapse, and distal axon, indicating enrichment across multiple levels of cellular components, including basic synaptic structural units (presynaptic membrane, postsynaptic membrane, and dense zone), neuronal subcellular structures (distal axon), and genetic foundations (chromosomal regions). In the MF category, significant enrichment was observed in terms such as channel activity and GTPase regulator activity. This suggests that the targets may function primarily through the following mechanisms: mediating transmembrane transport of substances (ions, metals, small molecules) as channels or transporters and regulating the activity of other enzymes (e.g., GTPases) as regulatory factors. KEGG pathway enrichment analysis showed that the targets were mainly associated with pathways such as Neuroactive ligand–receptor interaction, cAMP signaling pathway, and GABAergic synapse. These findings indicate that the targets may play potential roles in cell signal transduction (e.g., cAMP signaling pathway, calcium signaling pathway) and fine–tuned neural regulation (e.g., glutamatergic synapse, cholinergic synapse, GABAergic synapse).

### 2.8. Comprehensive Analysis of Molecular Mechanism of TPHP–Induced ASD Toxicity Based on Network Toxicology and Multiple Transcriptomic Data

Based on the intersection of functional pathways obtained from network toxicology analysis and GSE28521 transcriptomic data analysis with those from a transcriptomic analysis of E15 mouse forebrain samples under TPHP transgenerational exposure, one overlapping KEGG pathway was identified. The overlapping KEGG pathway obtained was GABAergic synapse. The core target *GAD1* identified in the aforementioned studies is believed to play a crucial role in the neurodevelopmental toxicity mechanism of TPHP−induced ASD through its mediation of the GABAergic synapse pathway.

### 2.9. Single–Cell Sequencing Analysis of Core Targets

We performed comprehensive quality control on the single–cell RNA sequencing dataset GSE203201 from the GPL24676 platform to exclude low–quality reads and technical artifacts. The 1500 most variable genes were selected for downstream analysis (Figure 4A). To address cellular heterogeneity, the UMAP (Uniform Manifold Approximation and Projection) method was employed, and three discrete cell types were annotated, including neuroepithelial cells, neurons, and iPS cells (Figure 4B,C). The core target *GAD1* exhibited a cell type–specific expression pattern. *GAD1* was primarily expressed and significantly upregulated in neurons, while it was downregulated in neuroepithelial cells and iPS cells (Figure 4D,E). These expression dynamics suggest that TPHP activates key neuronal cells through the *GAD1* target, thereby promoting the occurrence and development of ASD.

### 2.10. Virtual Knockout and Gene Enrichment Analysis of Core Target GAD1

To further validate the regulatory role of *GAD1* in ASD, we performed a simulated knockout analysis of the core target *GAD1* within the ASD group using the scTenifoldKnk algorithm based on the GSE203201 single–cell transcriptomic dataset, aiming to assess its perturbation effects under ASD conditions. A comprehensive analysis of Z–scores and FC values revealed that the top 10 genes most significantly and strongly affected by *GAD1* knockout included *STMN2*, *RTN1*, *LINC01551*, *INA*, *FTL*, *MLLT11*, *KIF5C*, *PCSK1N*, *SCG3*, and *DCX*. These genes all exhibited significantly elevated Z–scores and FC values. Subsequently, the significant DEGs following *GAD1* knockout were visualized using a volcano plot (Figure 4F), while the top 20 most significantly altered genes were visualized using a bar chart (Figure 4G).

To further explore the functional and pathway changes following *GAD1* knockout, we performed GO and KEGG functional pathway analysis on the significant DEGs after *GAD1* knockout. Using an adjusted *p*-value < 0.05 as the threshold, we obtained 104 significant GO terms (including 53 BP terms, 35 CC terms, and 16 MF terms) and one KEGG pathway. The top three GO terms from each of the three categories and the one KEGG signaling pathway were selected for visualization (Figure 4H). In the BP category, the targets were primarily enriched in terms such as synapse organization, axonogenesis and axon development—indicating that the significant DEGs after *GAD1* knockout have potential roles in neuronal structural development processes like synapse organization, axonogenesis and axon development. In the CC category, the targets were mainly enriched in terms like neuronal cell body, distal axon and growth cone—suggesting that these targets are enriched in cellular components such as neuronal cell bodies, distal axons and growth cones, playing important roles in nervous system development. In the MF category, significant enrichment was observed in terms such as tubulin binding, enzyme inhibitor activity and endopeptidase inhibitor activity. This indicates that the targets are importantly associated with enzyme inhibitors and tubulin binding. KEGG pathway enrichment analysis showed that the targets were primarily enriched in the cholesterol metabolism pathway. Cholesterol is not only a core structural component of neuronal cell membranes and myelin sheaths but also an essential regulator for synapse formation, neurotransmitter release, and signal transduction. The disruption of cholesterol metabolism homeostasis in the brain is a potential driver of a range of neurodevelopmental disorders. Comprehensive analysis indicates that the above results suggest that *GAD1* knockout may have a significant impact on neuronal structural development, such as synapse organization, axonogenesis, and axon development, as well as on the disruption of cholesterol metabolism homeostasis in the brain.

### 2.11. Molecular Docking Analysis of TPHP with GAD1 Protein

In summary, *GAD1* is a toxicological target involved in TPHP–induced ASD initiation and progression. To evaluate the interaction between TPHP and the core target protein GAD1, and to further validate that *GAD1* serves as a toxicological target in TPHP–induced ASD, molecular docking analysis was performed using AutoDockTools 1.5.7 software. The molecular docking binding energy between TPHP and the GAD1 protein was −7.9 kcal/mol (Figure 5A). A binding energy less than −4.25 kcal/mol indicates that the ligand and receptor have a certain binding activity, while a value less than −5.0 kcal/mol signifies good binding activity. The results demonstrate that TPHP exhibits superior binding activity with GAD1 protein. As molecular docking cannot account for the flexibility of protein structures, temperature, pressure, and solvent effects, subsequent molecular dynamic (MD) simulations were conducted on the TPHP−GAD1 complex to further explore the stability of the protein–ligand interaction.

### 2.12. Molecular Dynamic Simulation of TPHP–GAD1 Complex

To further explore the stability of the protein–ligand interaction, we performed a 100 ns molecular dynamic (MD) simulation of the TPHP–GAD1 complex (Figure 5B–H). The RMSD curve reflects the fluctuation in the protein conformation [33]. The average RMSD of the TPHP–GAD1 complex was 0.21 ± 0.02 nm, with small fluctuations, indicating that the complex rapidly reached equilibrium and remained stable during the 100 ns simulation. The RMSD of the GAD1 protein was 0.15 ± 0.02 nm, and the RMSD of the ligand TPHP was 0.15 ± 0.03 nm. Both values were below the typical instability threshold (>0.3 nm), suggesting that the binding of TPHP to GAD1 protein does not cause a drastic perturbation of the protein conformation and promotes the increased rigidity of the TPHP–GAD1 complex (Figure 5B). RMSF can reflect the fluctuation in the complex at the residue level [34]. The average RMSF value of the GAD1 protein was 0.11 ± 0.06 nm, indicating that most residues exhibited low fluctuation during the simulation. A low RMSF reflects the stabilizing effect of TPHP–GAD1 complex formation (Figure 5C). Hydrogen bonds are strong non–covalent interactions between proteins and ligands, and their number reflects the strength of the interaction. Throughout the simulation, the number of hydrogen bonds in the TPHP–GAD1 complex ranged from 0 to 2 (average 0.27 ± 0.46), with a maximum of 2. Hydrogen bonds help enhance the specific binding of the TPHP–GAD1 complex, and the hydrogen bonds between the ligand and protein contribute to maintaining the stability of the TPHP–GAD1 complex (Figure 5D). Rg reflects the tightness of binding and the degree of system constraint, as well as the degree of protein folding, and is used to evaluate the binding compactness of the complex. A higher Rg value is associated with an increased likelihood of generating flexible ligands, so a higher Rg value indicates lower stability, while a lower Rg value suggests a structurally dense and tightly packed system [35]. During the simulation, the average Rg value of the TPHP–GAD1 complex was 2.87 ± 0.01 nm, indicating that the overall protein structure is compact and stable. This stable Rg value reflects that the TPHP–GAD1 complex maintained its folded state in a dynamic environment, which is conducive to the long–term binding of the ligand TPHP (Figure 5E). SASA reflects changes in the protein surface exposed to the solvent and can indicate protein folding and stability [36]. During the simulation, the average SASA value of the GAD1 protein was 359.19 ± 4.39 nm^2^. The small standard deviation indicates stable surface exposure without significant hydrophilic or hydrophobic changes. The constant SASA value suggests that the binding of the ligand TPHP may partially shield certain solvent−exposed regions, optimizing the solvation environment of the GAD1 protein and enhancing the hydrophobic interactions of the TPHP–GAD1 complex (Figure 5F). The Gibbs free energy landscape (FEL) reveals the stability of the complex [37]. In the FEL of the TPHP–GAD1 complex (Figure 5G,H), the TPHP–GAD1 complex resides in a relatively stable conformational state. Calculating the binding free energy can validate the strength of intermolecular interactions within the protein–ligand complex [38]. To quantify binding affinity, we employed the molecular mechanics Poisson–Boltzmann surface area (MM–PBSA) method to calculate the binding free energy (Table 1). The results showed that the van der Waals energy contribution (ΔVDWAALS) was −41.20 ± 0.69 kcal/mol, the electrostatic energy contribution (ΔEelec) was −8.23 ± 0.28 kcal/mol, the non−polar solvation energy (ΔEsurf) was −5.27 ± 0.03 kcal/mol, and the polar solvation energy (ΔEGB) was 30.73 ± 0.94 kcal/mol. The total gas−phase free energy (ΔGgas) was −49.43 ± 0.74 kcal/mol, the solvation free energy (ΔGsolvation) was 25.45 ± 0.94 kcal/mol, and the final total binding free energy (ΔTotal) was −23.98 ± 1.20 kcal/mol. This indicates a strong interaction between the ligand TPHP and the protein GAD1, demonstrating good binding affinity. All the above analyses indicate the stability of the protein GAD1–ligand TPHP interaction, further confirming the potential of TPHP in regulating protein GAD1–related biological processes.

### 2.13. Construction of AOP for TPHP–Induced ASD

Based on a comprehensive analysis of the above research results, we constructed a novel AOP framework describing TPHP–induced ASD by systematically integrating molecular targets and signaling networks. This AOP framework indicates that TPHP alters the expression and activity of the core gene *GAD1*, which in turn modulates the GABAergic synapse signaling pathway. This leads to an imbalance between excitatory and inhibitory neurotransmission in the brain, resulting in neurodevelopmental toxicity and subsequently inducing and exacerbating the occurrence and progression of ASD (Figure 6). The OECD handbook was used to evaluate the biological plausibility and empirical support of the AOP framework. To assess the weight of evidence (WoE) for our AOP, we retrieved high−confidence evidence from PubMed and Web of Science (Tables S4 and S5). In summary, this study demonstrates that the *GAD1*–mediated AOP framework can be employed to investigate the risk of ASD induced by TPHP exposure, providing new insights into the occurrence and development of ASD triggered by the environmental chemical TPHP.

### 2.14. Identification of Neurodevelopmental Toxicity Risks of Aryl OPFRs Based on Batch Molecular Docking Using AutoDock Vina Software

We used AutoDockTools 1.5.7 software to perform a batch molecular docking analysis of 85 OPFRs with the GAD1 protein to evaluate their interactions and binding potential, ultimately obtaining the binding energies from molecular docking (Table S6). The results of molecular docking showed that the top five OPFRs with the strongest binding ability to the GAD1 protein were T4NPPP, DP4PPP, B24DTBPPTDP, BDP, and PBDMPP, with binding energies of −10.1 kcal/mol, −9.2 kcal/mol, −9.1 kcal/mol, −8.9 kcal/mol, and −8.9 kcal/mol, respectively (Figures S1–S5). This indicates that these five OPFRs have good interaction and binding capabilities with the GAD1 protein. They may act on the *GAD1* target, disrupting the signaling pathways regulated by *GAD1*, thereby inducing neurodevelopmental toxicity and promoting the occurrence and progression of neurodevelopmental disorders, including ASD. Based on the PubChem database, we further examined the chemical structures of these five OPFRs. Notably, all five OPFRs belong to the category of OPFRs containing phenyl ring substituents (i.e., aryl OPFRs). This suggests that, compared to other structural groups, aryl OPFRs containing phenyl ring substituents exhibit higher binding energies with the GAD1 protein, indicating a more pronounced potential impact of aryl OPFRs on neurodevelopment. The above research findings elucidate the neurodevelopmental toxic effects of aryl OPFRs and provide valuable chemical references for the neurodevelopmental toxicity assessment of OPFRs.

## 3. Discussion

Organophosphorus flame retardants (OPFRs), which have become the main flame retardants in indoor environments as an alternative to brominated flame retardants [39], have gradually been recognized as endocrine–disrupting chemicals (EDCs) [40]. Exposure to endocrine–disrupting chemicals (EDCs) may adversely affect physiological processes such as reproduction, fetal/child development, metabolism, and neurological function [41]. TPHP, as a representative of classical OPFRs, is widely used and has been detected at high frequency in a variety of environmental media [42,43] and human samples [7,44]. In recent years, several studies have indicated that TPHP exposure can induce adverse neurodevelopmental outcomes [14,45]. Although ASD has a significant genetic etiology, environmental factors (e.g., toxins, pesticides, infections, and medications) increase susceptibility to ASD [46], and it has been reported that 40–50% of the etiology of ASD can be attributed to environmental factors [26], and considering the global disease burden of ASD, this study focused on TPHP exposure and ASD disease. The frontal lobe cortex is closely related to neural activities such as emotion, decision–making, and social cognition [47]. It is a key region for integrating functions and social abilities, and its abnormalities are believed to be associated with ASD [48,49]. Social behavioral deficits are a hallmark of ASD, and the involvement of the frontal cortex in social behavior has been well–established [50]. This study further focuses on investigating the toxicological effects of TPHP exposure in the frontal cortex tissue on ASD.

Traditional toxicity assessment methods based on animal models are costly, time–consuming, and ethically challenging, making them ineffective in keeping pace with the increasing number of chemicals and their toxicity evaluations [51]. With the proposal of the 21st–century toxicology vision and the rapid development of computer science and technology, computational toxicology has emerged as a promising alternative method for predicting the toxic potential of chemicals. It is now widely applied in chemical toxicity studies, utilizing computer technology to investigate the toxic effects of chemicals. This approach offers advantages such as high efficiency, low cost, high–throughput screening, and greater ethical benefits [52,53]. Computational toxicology encompasses various techniques, including ADMET prediction for chemicals, network toxicology, molecular docking, and molecular dynamic simulations, and integrates advanced algorithms such as virtual knockout for comprehensive multi–dataset analysis. This has brought a transformative paradigm to the field of toxicology research [54]. Our study, based on these computational toxicology techniques, elucidates the complex toxic mechanisms of TPHP–induced ASD, identifies potential intervention targets, and ultimately reveals the key molecular events in the process of TPHP–induced ASD.

In summary, this study first evaluated the physicochemical properties and ADMET characteristics of TPHP. Subsequently, through a network toxicology analysis of TPHP and ASD, as well as transcriptomic analysis using the GEO database, to thoroughly investigate and validate the complex toxic mechanisms of TPHP–induced ASD, we performed transcriptomic analysis on E15 mouse forebrain samples from transgenerationally exposed TPHP. Integrating network toxicology, GEO database analysis, and a transcriptomic analysis of transgenerationally exposed E15 mouse forebrain samples, the results clearly identified that the dysregulation of the GABAergic synapse pathway likely plays a crucial role in TPHP–induced ASD. The *GAD1* target plays a significant role in the dysregulation of the GABAergic synapse pathway and was therefore selected for subsequent analysis using the GSE203201 single–cell dataset. The analysis revealed that TPHP activates neuronal cell subpopulations through the *GAD1* target, thereby promoting the occurrence and progression of ASD. To further validate the impact of significantly downregulated *GAD1* expression during TPHP exposure on ASD, a simulated gene knockout of the *GAD1* target was performed. The significant DEGs following *GAD1* knockout showed potential effects on biological processes such as synapse organization, axonogenesis, and axon development, fully confirming that the *GAD1* target is a key molecular event in TPHP–induced ASD. Finally, molecular docking and molecular dynamic simulations thoroughly confirmed the stable interaction between TPHP and GAD1 protein, further supporting the role of *GAD1* as a key target in the progression of TPHP–induced ASD. Subsequently, using the GAD1 protein as the receptor, batch molecular docking was performed with 85 organophosphorus flame retardants (OPFRs) widely detected in human and biological matrices. The results indicated that aryl–OPFRs are disruptors that target *GAD1* to produce neurodevelopmental toxicity.

Glutamic acid decarboxylase 67 (*GAD1*) is associated with the pathophysiology of ASD [55]. One study provided evidence that prenatal valproic acid (VPA) exposure resulted in the dysregulated expression of *GAD1* in the prefrontal cortex and cerebellum [56], and another showed the same dysregulation in autopsy samples from the brains of ASD patients [57]. In mammals, two isoenzymes, glutamate decarboxylase 1 (*GAD1*, 67 kDa) and glutamate decarboxylase 2 (*GAD2*, 65 kDa), catalyze the synthesis of γ–aminobutyric acid (GABA) from glutamate [58]. In the mature brain, GABAergic synapses, the predominantly inhibitory type of synapses in the CNS, have a central function through the release of the inhibitory neurotransmitter γ–aminobutyric acid (GABA). GABA, as the major inhibitory neurotransmitter in the CNS, reduces neuronal activity, prevents the over–excitation of nerve cells, and plays an important role in maintaining the excitatory–inhibitory balance (E/I balance) of the brain [59]. In the healthy adult brain, the balance between excitatory and inhibitory (E/I) states is tightly regulated, and in contrast to GABA, glutamate is the major excitatory neurotransmitter, and decreased inhibition, increased excitability, or both result in an E/I imbalance, and E/I imbalance in the brain is a common mechanism for neurodevelopmental disorders and psychiatric disorders [60]. Multiple studies have confirmed that the disruption of GABAergic signaling and the resulting excitatory–inhibitory imbalance (E/I imbalance) are associated with the pathophysiology of ASD and that there is an abnormality in the regulation of GABAergic signaling in ASD [56]. Experimental animal studies have demonstrated the reduced expression of GABA levels in rat models of ASD [46]. Chalkiadaki et al. developed a human brain organoid model derived from induced pluripotent stem cells (iPSCs), employing targeted genome editing to eliminate the protein expression of ASD risk genes. Subsequent proteomic analysis revealed disruptions in the glutamatergic/GABAergic (γ–aminobutyric acid) synaptic pathways and neurodevelopment [61]. Data from clinical studies also show that GABAergic neurotransmission is reduced in the brains of ASD patients [62]. The functional properties of GABA in the immature brain are very different from those in the adult brain, where GABA depolarizes target cells and triggers calcium inward flow in the embryonic and perinatal periods. GABA–mediated calcium signaling regulates a variety of developmental processes such as cell proliferation, migration, differentiation, synapse maturation, and cell death [63]. It is noteworthy that a gene knockout study demonstrated that *Gad1* knockout mice exhibited disruption in GABA levels [64]. Another *GAD1* gene knockout study using immunohistochemistry also showed a substantial reduction in GABA synthesis in *Gad1* gene knockout rats [58]. Furthermore, the active region of *GAD1* on human chromosome 2q21–q33 is a susceptibility locus for ASD. *GAD1* may serve as a potential marker for GABAergic abnormalities in ASD [56].

Recent research breakthroughs have revealed the central role of glial cells in the regulation of GABAergic synapses and the excitatory–inhibitory (E/I) balance [65]. Based on the above analysis, in the immature brain during the early postnatal period, due to the high intracellular chloride ion concentration primarily maintained by the NKCC1 transporter, GABA activates GABA_A receptors, leading to chloride efflux and producing a depolarizing effect. This plays an important role in promoting neuronal migration and synapse formation. As the brain develops, the expression of the KCC2 transporter increases, reducing the intracellular chloride ion concentration. Consequently, GABA transitions to a hyperpolarizing inhibitory neurotransmitter, thereby maintaining the balance between excitation and inhibition (E/I balance) in the brain [66]. Glial cells play a crucial role in maintaining chloride ion homeostasis, with astrocytes and microglia regulating the intracellular chloride concentration that determines the effect of GABA [67,68]. The disruption of chloride ion homeostasis leads to dysregulation in the conversion between depolarization and hyperpolarization, which may result in an imbalance between brain excitation and inhibition [69]. When *GAD1* expression or function is abnormal, it leads to weakened GABAergic inhibitory signaling, disrupts the E/I balance, and causes excessive neuronal network excitation. This, in turn, releases a large amount of signaling molecules such as glutamate and ATP, which are key activation signals for microglia [70]. Therefore, *GAD1* dysfunction can be regarded as an upstream perturbation. By disrupting neurotransmitter homeostasis, it indirectly triggers the abnormal activation of microglia and neuroinflammatory responses, which in turn leads to the dysregulation of intracellular chloride ion concentration in neurons, exacerbating the imbalance between brain excitation and inhibition.

The neurodevelopmental toxicity of aryl OPFRs has been confirmed by relevant studies [71,72]. Epidemiological studies have shown a significant association between prenatal OPFR exposure and adverse neurodevelopmental outcomes in offspring. For example, the concentrations of various OPFR metabolites (including metabolites of aryl OPFRs) in maternal urine are associated with lower neurodevelopmental scores in children. Even during intrauterine development, exposure to aryl OPFRs may negatively impact future cognitive, motor, and behavioral abilities [73].

Animal experiments have further revealed the sex–specific and long–term nature of their neurodevelopmental toxicity. A study using wild–type mice exposed to an OPFR mixture (containing aryl components) or a sesame seed oil vehicle from gestational day 7 to postnatal day 14 demonstrated that perinatal OPFR exposure has sex–and exposure–dependent effects on locomotor and anxiety–like behaviors in adult mice [74]. Another study in Wistar rats demonstrated that perinatal (gestational and lactational) exposure to an OPFR mixture (containing aryl components) significantly disrupted adult hippocampal neurogenesis, with sex–specific effects. In male rats, exposure primarily resulted in a reduction in the number of neural progenitors and new/immature neurons, as well as a decrease in dentate gyrus volume. In contrast, female rats exhibited an increase in the number of neural progenitors and a decrease in the number of new/immature neurons [75]. These findings not only confirm the long–term damage of aryl–OPFRs to key brain plasticity regions but also emphasize the importance of considering sex as a biological variable in neurodevelopmental toxicology assessments.

In summary, under TPHP exposure, *GAD1* may be a key target for GABAergic abnormalities leading to an imbalance between brain excitation and inhibition in ASD. On one hand, as the rate–limiting enzyme for GABA synthesis, the abnormal expression or function of *GAD1* leads to weakened GABAergic inhibitory signaling, disrupting the brain’s excitation–inhibition balance. On the other hand, *GAD1* dysfunction can be regarded as an upstream perturbation that triggers the abnormal activation of microglia and neuroinflammatory responses, resulting in the dysregulation of intracellular chloride ion concentration and exacerbating the excitation–inhibition imbalance. Aryl–OPFRs exhibit good binding affinity to the active site of the GAD1 protein, acting as disruptors of neurodevelopment, which provides valuable chemical references for the neurodevelopmental toxicity assessment of OPFRs.

Although this study employed comprehensive computational methods, it still has some limitations. Most of the proposed mechanisms are derived from computational analyses of public transcriptomic datasets, with limited data from in vivo and in vitro studies, necessitating further experimental validation—which is crucial for further elucidating the toxicological effects of TPHP in the induction and development of ASD. Furthermore, while our analysis focused on pathways related to the *GAD1* target, other mediators or cofactors may also warrant further investigation. The correspondence between the predicted molecular interactions and actual human exposure to TPHP remains uncertain; future studies should emphasize research on TPHP toxicokinetics, biomonitoring, and dose–response analysis to better understand the association between TPHP exposure levels and related health risks. Validation experiments for *GAD1* should also be integrated with toxicokinetics, biomonitoring, and population–based analyses to comprehensively elucidate the health risks of TPHP.

## 4. Materials and Methods

### 4.1. Evaluation of ADMET Characteristics of TPHP

The ADMETlab 3.0 database (https://admetlab3.scbdd.com/) comprehensively covers the absorption, distribution, metabolism, excretion, and toxicity (ADMET) properties of compounds and is widely used for screening the ADMET characteristics and biological activities of compounds [76]. The chemical structure and Simplified Molecular Input Line Entry System (SMILES) notation of TPHP were retrieved from the PubChem database (https://pubchem.ncbi.nlm.nih.gov/). The toxicity data of TPHP were retrieved from the ADMETlab 3.0 database. The absorption properties of TPHP were assessed by evaluating its permeability in Madin–Darby Canine Kidney (MDCK) cells and Human Colorectal Adenocarcinoma Cells (Caco–2). The distribution properties of TPHP were evaluated using its plasma protein binding (PPB) value and the fraction unbound in plasma (Fu) value. The metabolic properties of TPHP were assessed by evaluating its probability of being a substrate for CYP1A2, CYP2C19, CYP2C9, CYP2D6, CYP3A4, and CYP2B6. The excretion properties of TPHP were evaluated using its clearance (CL) value. Finally, the probability of TPHP crossing the blood–brain barrier (BBB) was assessed. The absorption, distribution, metabolism, excretion, and toxicity characteristics of TPHP were comprehensively evaluated [54,77].

### 4.2. Collection of TPHP−and ASD−Related Targets

TPHP−related targets were identified using the CTD (Comparative Toxicogenomics Database) (URL: https://ctdbase.org/) and SEA (Similarity Ensemble Approach) database (URL: https://sea.bkslab.org/), both accessed on 28 February 2026. In the CTD database, the “Chemicals” category was selected, and the search term “Triphenyl phosphate” was used to obtain TPHP−related gene targets. The SMILES notation of TPHP was obtained from the PubChem database (URL: https://pubchem.ncbi.nlm.nih.gov/). The SMILES notation of TPHP was input into the SEA database, with the species set to “*Homo sapiens*,” to obtain TPHP–related gene targets. The targets were merged, and duplicates were manually removed to obtain the final set of TPHP–related targets. ASD–related targets were obtained from the OMIM (Online Mendelian Inheritance in Man) database (URL: https://omim.org/) and the GeneCards database (URL: https://www.genecards.org/), both accessed on 26 February 2026. Both databases were searched using the term “Autism Spectrum Disorder” to obtain ASD–related gene targets. In the GeneCards database, targets with a “Protein Coding” classification and a “Relevance score” greater than 10 were included in subsequent analyses. All ASD–related genes from the OMIM database were included in the subsequent analysis. The ASD–related target genes involving the human species retrieved from the above two databases were combined, and duplicates were manually excluded to identify potential ASD–related targets. The intersection of ASD–related targets and TPHP–related targets was presented as potential targets for TPHP–induced ASD [78]. 

### 4.3. Construction of Interaction Network of Potential Targets of ASD Induced by TPHP

To further investigate the potential targets of TPHP–induced ASD, the aforementioned potential targets were submitted to the STRING database (https://cn.string-db.org/) to construct a protein–protein interaction (PPI) network [79]. The analysis was limited to the species “Homo sapiens.” To study the core regulatory mechanisms of TPHP−induced ASD, a threshold of the “highest confidence (0.900)” was selected to screen for highly credible interacting core targets. Target genes that did not interact with any other proteins were removed. Subsequently, the edges, nodes, and degree values of the target genes were examined to construct the PPI network. The topological parameters of the aforementioned targets were calculated using the network analysis tool of Cytoscape 3.8.0 software (https://cytoscape.org/) [80]. Based on the condition that the degree value is greater than 5, the potential core targets of TPHP−induced ASD were further screened.

### 4.4. Microarray Data Sources and Differential Expression

Using the search terms “ASD” and “Frontal cortex”, gene expression data related to ASD were retrieved from the GEO (Gene Expression Omnibus) database (https://www.ncbi.nlm.nih.gov/geo/) (datasets GSE28521 [81] from GPL6883 and GSE113834 [82] from GPL15207). URL (accessed on 28 February 2026). The frontal cortex tissue dataset was selected from the GSE28521 dataset of GPL6883, which included the control group (*n* = 16) and the ASD group (*n* = 16), and the GSE113834 dataset was derived from prefrontal cortex samples, divided into two groups, including the control group (*n* = 12) and the ASD group (*n* = 15), and the dataset was preprocessed with background adjustments and normalization for evaluation. For the GSE28521 and GSE113834 datasets, information such as patient age, sex, RNA integrity number (RIN), and Postmortem interval (PMI) can be found in the Supplementary Materials (Table S3). The GSE113834 dataset was used for subsequent analyses as a validation dataset for the GSE28521 dataset. The GSE28521 dataset was screened for differentially expressed genes (DEGs) between the control and ASD groups in each dataset by the “limma” package [83]. The criteria for identifying differentially expressed genes (DEGs) were defined as follows: |log2FC(Fold change)| ≥ 0.585 and *p*-value < 0.05.

### 4.5. Network Toxicology and Transcriptomic Integrated Analysis: Target Intersection and Functional Pathway Analysis

The potential targets of TPHP–induced ASD and the molecular mechanism of its toxic effects were analyzed by network toxicology and transcriptomics. GO (Gene Ontology) and KEGG (Kyoto Encyclopedia of Genes and Genomes) enrichment analyses of the intersection targets from network toxicology and GSE28521 DEGs were conducted to explore their biological roles and molecular mechanisms [84,85], with an adjusted *p*-value less than 0.05 considered statistically significant. 

### 4.6. Differential Expression Analysis and Diagnostic Accuracy Evaluation of Core Targets

Based on the GEO datasets GSE28521 and GSE113834 (which contain transcriptomic data from ASD patients and normal controls, with GSE113834 serving as the validation set for GSE28521), we first analyzed and visualized the expression levels of the core targets in these two datasets. Targets with a *p*-value < 0.05 were considered significantly differentially expressed. The ROC (receiver operating characteristic) curve represents the true positive rate versus the false positive rate of the core targets across a range of classification thresholds, providing a visual representation of their predictive effectiveness. Based on the two aforementioned datasets, we constructed single–gene ROC curves using the “pROC” package to further assess the diagnostic potential of the identified core targets [86]. Finally, the diagnostic accuracy of the ROC curves was assessed by calculating the AUC (Area under the curve).

### 4.7. Establishment of TPHP–Exposed Mouse Cohorts

Specific pathogen–free (SPF) grade C57BL/6J mice were utilized for this study. Female mice were 8 weeks old, and male mice were 7 weeks old at the time of purchase from Spelford (Beijing, China) Biotechnology Co. All experimental C57BL/6J mice were housed in the animal experiment center at the Yanhu campus of Ningxia Medical University in the Animal Research Facility. This study received ethical approval from the Ningxia Medical University Animal Care and Use Committee (NO. IACUC-NYLAC-2024-001). The mice were maintained under controlled environmental conditions: a temperature range of 20–25 °C, constant humidity, with ad libitum access to food and water. For breeding, at 17:00 each afternoon, two female mice and one male mouse were randomly assigned to co–habit in the same cage. The following morning at 08:00, the male mouse was removed. Female mice were then examined for the presence of a vaginal plug as an indicator of successful mating and potential pregnancy. Female mice exhibiting signs of conception were designated as at gestational day 0, with the corresponding embryonic day 0 recorded. Regular measurements of body weight and abdominal circumference were subsequently taken for these pregnant females. Based on the correspondence between human and mouse lifespans and the modified one–generation toxicology study design from the National Toxicology Program [87], and with reference to the estimated daily intake levels of OPFRs and TPHP [88,89], the doses were determined using a C57BL/6J mouse model following cross–species conversion based on body surface area while also considering median lethal dose data and observed adverse effects. The low dose was set at 0.1 mg/kg bw/day. Since the LD50 for TPHP is not reported in current studies, the medium and high doses were determined by sequentially increasing the low dose by a factor of 10, resulting in 1 mg/kg bw/day and 10 mg/kg bw/day, respectively. These dose levels are at or below previously reported exposure doses in toxicological studies. Accordingly, pregnant dams in the experimental groups were exposed to TPHP at three dose gradients (0.1 mg/kg, 1 mg/kg, and 10 mg/kg), while the control group was exposed to the solvent vehicle. The vehicle was prepared using DMSO, PEG300, Tween 80, and saline. TPHP exposure via oral gavage commenced on gestational day 6 (post–implantation), and TPHP was administered once daily until postnatal day 21. For subsequent transcriptomic analysis, embryonic day 15 offspring from dams in the 10 mg/kg TPHP exposure group (*n* = 3) and the solvent vehicle control group (*n* = 3) were selected.

### 4.8. Transcriptomic Analysis of Embryonic Day 15 Mouse Forebrain Samples

On embryonic day 15, the dams were euthanized under isoflurane anesthesia, and the forebrain tissues of the offspring were collected. The samples were snap–frozen in liquid nitrogen for 30 min and then transferred to a −80 °C freezer for long–term storage. Transcriptomic analysis was subsequently performed on the forebrain samples from the embryonic day 15 offspring of the experimental group (*n* = 3) and the control group (*n* = 3). Total RNA was extracted using TRIzol reagent according to the manufacturer’s instructions. RNA purity and concentration were assessed using a NanoDrop 2000 spectrophotometer (Thermo Scientific, Wilmington, DE, USA), and RNA integrity was evaluated using an Agilent 2100 Bioanalyzer (Agilent Technologies, Santa Clara, CA, USA). Transcriptome libraries were constructed using the VAHTS Universal V10 RNA–seq Library Prep Kit (Premixed Version) following the manufacturer’s protocol. Transcriptome sequencing and analysis were performed by Shanghai Ouyi Biotechnology Co., Ltd. (Shanghai, China). Libraries were sequenced on the Illumina NovaSeq 6000 platform to generate 150 bp paired–end reads. Raw reads in FASTQ format were processed using fastp 0.20.1 software to remove low–quality reads, yielding clean reads for subsequent analyses. HISAT2 2.1.0 software was used for reference genome alignment, and gene expression levels (FPKM) were calculated. Read counts per gene were obtained using HTSeq–count. Principal component analysis (PCA) and visualization were performed using R (v 4.5.0) based on gene counts to evaluate biological replicates. Differential expression analysis was conducted using DESeq2. Differentially expressed genes (DEGs) were defined based on the following criteria: |log2FC| ≥ 0.585 and adjusted *p*-value < 0.05. To further explore the potential functional changes in the identified DEGs, Gene Ontology (GO) functional enrichment analysis and Kyoto Encyclopedia of Genes and Genomes (KEGG) pathway enrichment analysis were performed.

### 4.9. Integrated Analysis of Network Toxicology and Multiple Transcriptomic Datasets

Based on network toxicology analysis, GSE28521 transcriptomic data analysis, and transcriptomic analysis of E15 mouse forebrain samples from TPHP transgenerational exposure, overlapping pathways were screened to identify those playing a significant role in the neurodevelopmental toxicity mechanisms of TPHP–induced ASD.

### 4.10. Analysis of Single–Cell RNA Sequencing Datasets

In this study, the scRNA–seq dataset GSE203201 [90] was obtained from the GEO database. We extracted samples from the GSE203201 dataset corresponding to days 6, 12, 30, 60, and 100 of brain organoid culture in vitro, including both control (*n* = 7) and experimental (*n* = 7) groups. In single–cell RNA–seq data processing, high−quality cells were selected based on a mitochondrial gene percentage below 15% and the detection of more than 50 genes. The Seurat workflow was employed for data integration and normalization. Normalization was performed using the “LogNormalize” method, and the top 1500 highly variable genes were identified with the “FindVariableFeatures” function. Dimensionality reduction was carried out by principal component analysis (PCA), and batch effects across samples were corrected using the Harmony package [91,92]. Cell clustering was performed using the “FindClusters” function. Cell–type annotation was then conducted based on the “SingleR” package and the expression of classical cell marker genes. 

### 4.11. Simulated Gene Knockout and Gene Enrichment Analysis

To explore the regulatory logic centered on *GAD1* at single–cell resolution and investigate the potential impact of cell–cell communication outcomes, we performed network−based simulated gene knockout using the “scTenifoldKnk” R package version 1.0.3 and further predicted specific gene functions [91,93]. When the parameter “qc” is set to TRUE, quality control excludes cells with gene counts < 1000 or mitochondrial transcript proportion > 10%. The perturbation effect is quantified by a distance based on manifold alignment, converted into a Z–score normalized perturbation score, and used to rank downstream effects at the gene level. Genes are sorted according to their perturbation Z–scores, and the inferred directionality is interpreted as a predictive decrease/increase in regulatory activity (network influence), rather than directly measured differential expression [94]. Based on an adjusted *p*-value < 0.05, we screened for genes potentially affected by *GAD1* virtual knockout. To gain deeper insights into their potential functional changes, we further performed GO functional enrichment analysis and KEGG pathway enrichment analysis [95]. 

### 4.12. Molecular Docking

The molecular structure of TPHP was obtained from the PubChem database (http://pubchem.ncbi.nlm.nih.gov/). The energy minimization of the small molecule was performed using Chem3D 23.0 software, and the structure was subsequently converted into the mol2 format. The three–dimensional structures of core target proteins were retrieved from the Protein Data Bank (PDB) (http://www.rcsb.org/). Water molecules and ligands were removed using PyMOL 2.5.8 software, and the proteins were converted into the pdbqt format using AutoDockTools 1.5.7. The docking grid box parameters were set as follows: center_x = 2.893, center_y = 0.428, center_z = 25.42; size_x = 96.0, size_y = 54.0, size_z = 90.0. Molecular docking was then performed using Vina. Binding energy was used to evaluate the interaction between the ligand and the receptor: a binding energy less than 0 kcal/mol indicates spontaneous binding, while a binding energy lower than −5 kcal/mol signifies stable binding [96].

### 4.13. Molecular Dynamic Simulation

We performed molecular dynamic simulations of the TPHP–GAD1 complex using GROMACS software (version 5.1.5) [97,98]. The ligand topology file was generated using the AMBER force field with the ACPYPE script, whereas the AMBER99SB–ILDN force field was employed to generate the protein topology file. Prior to the commencement of molecular dynamic simulations, the system was neutralized by adding NaCl counterions. Subsequently, the complex system underwent energy minimization for 1000 steps, followed by 100 ps of equilibration simulations under both NVT and NPT ensembles. Each system was subjected to molecular dynamic (MD) simulations under periodic boundary conditions at 310 K and 1.0 bar for a duration of 100 ns. The minimum distance between the simulation box and the protein in the XYZ directions was set to 1.0 nm, with a time step of 2 fs. Energy minimization was performed using the steepest descent algorithm, and the cutoff distances for Coulomb and van der Waals interactions were set to 1.4 nm. In the molecular dynamic simulations, the system was placed within a triclinic lattice containing TIP3 water molecules. Analyses were performed on the RMSD (root mean square deviation), Rg (radius of gyration), SASA (solvent–accessible surface area), RMSF (root mean square fluctuation), HBonds (number of hydrogen bonds), and FEL (Gibbs free energy landscape).

### 4.14. Construction of AOP

Based on the comprehensive analysis of TPHP target data, ASD–related phenotypes, and gene–phenotype relationships, an AOP model for TPHP exposure–induced ASD occurrence and development was further developed. By mapping the molecular initiating event (MIE) and downstream key events (KEs), a mechanistic framework linking TPHP exposure to ASD disease outcomes was established, which contributes to a deeper understanding of the potential role of TPHP in the pathogenesis and progression of ASD [99]. Finally, the OECD handbook was utilized to assess the biological plausibility and empirical support of the AOP framework [100].

### 4.15. Batch Screening of OPFRs Based on Molecular Docking Methods

The SDF files of 85 OPFRs widely detected in human and biological matrices were obtained by downloading them from the PubChem database, further converting the SDF file format to mol2 format and conducting the energy minimization of small molecules using Chem3D 23.0 software. The 3D structure of the core protein GAD1 was obtained from the PDB database and converted to pdbqt format using AutoDockTools 1.5.7, and water molecules and ligands were removed using pymol software with docking box parameters of center_x = 2.893, center_y = 0.428, center_z = 25.42, size_x = 96.0, size_y = 54.0, and size_z = 90.0. The batch molecular docking of 85 OPFRs with the GAD1 protein was conducted using Vina, and binding energy was applied to screen OPFRs that exacerbate ASD neurodevelopmental toxicity via the *GAD1* target [32,101].

## 5. Conclusions

TPHP is a neurotoxicant characterized by high absorption, resistance to metabolism and excretion, and the ability to cross the blood–brain barrier. Under TPHP exposure, *GAD1* expression is significantly downregulated in frontal cortex tissue, activating the GABAergic synapse signaling pathway. This leads to an imbalance between brain excitation and inhibition, resulting in neurodevelopmental toxicity and inducing and exacerbating the occurrence and progression of ASD. In the neurodevelopmental toxicity assessment of OPFRs, aryl −OPFRs should be prioritized for consideration and given focused attention in subsequent research on OPFRs’ neurodevelopmental toxicity.

## Figures and Tables

**Figure 1:**
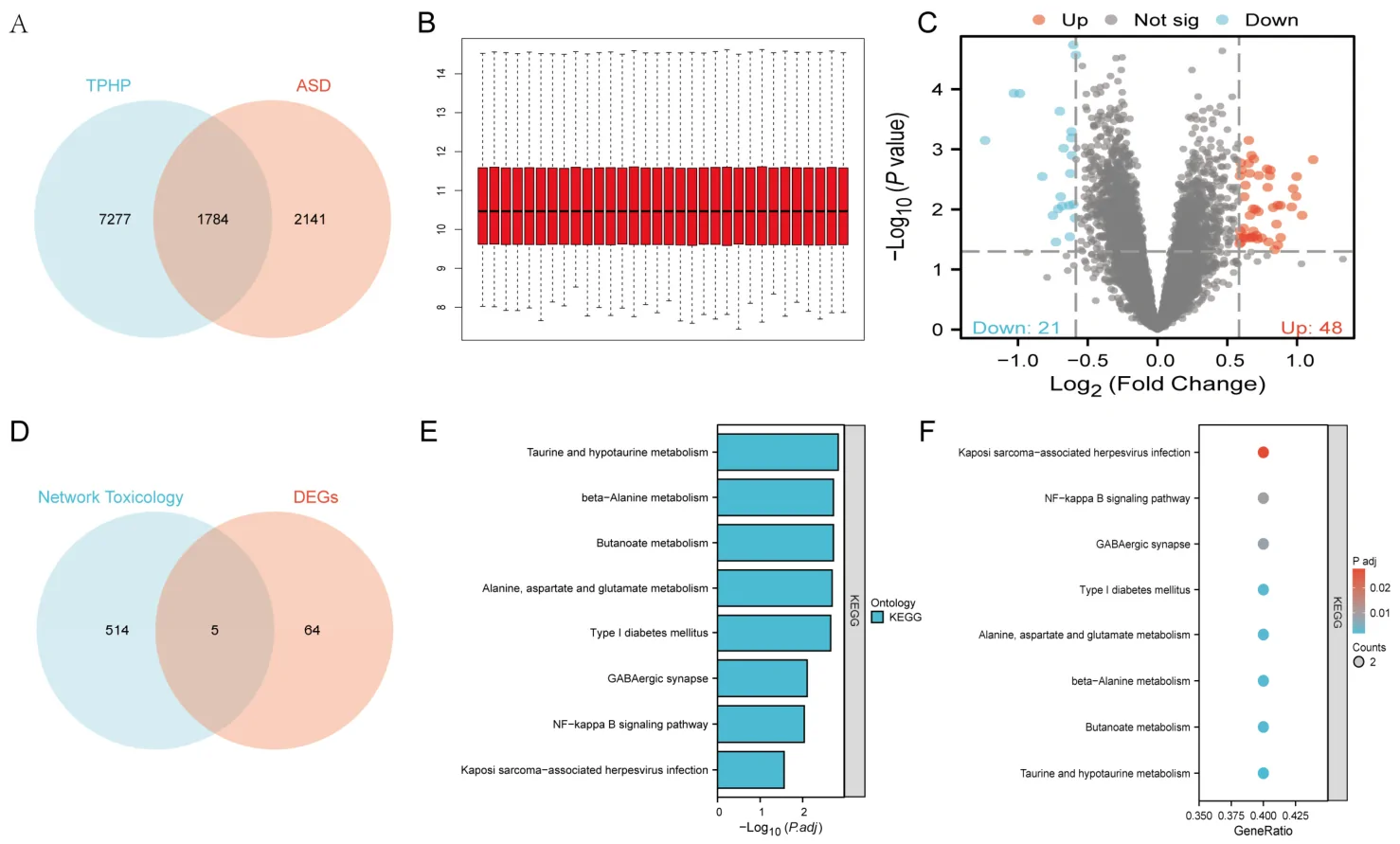
(**A**) Intersection of TPHP and ASD–related targets. (**B**) Comparability assessment of GSE28521 transcriptomic data. (**C**) Volcano plot of DEGs from GSE28521 dataset. (**D**) Intersection of targets from network toxicology, DEGs of GSE28521 dataset. (**E**) Bar plot of KEGG pathway enrichment analysis for intersected targets. (**F**) Bubble plot of KEGG pathway enrichment analysis for intersected targets.

**Figure 2:**
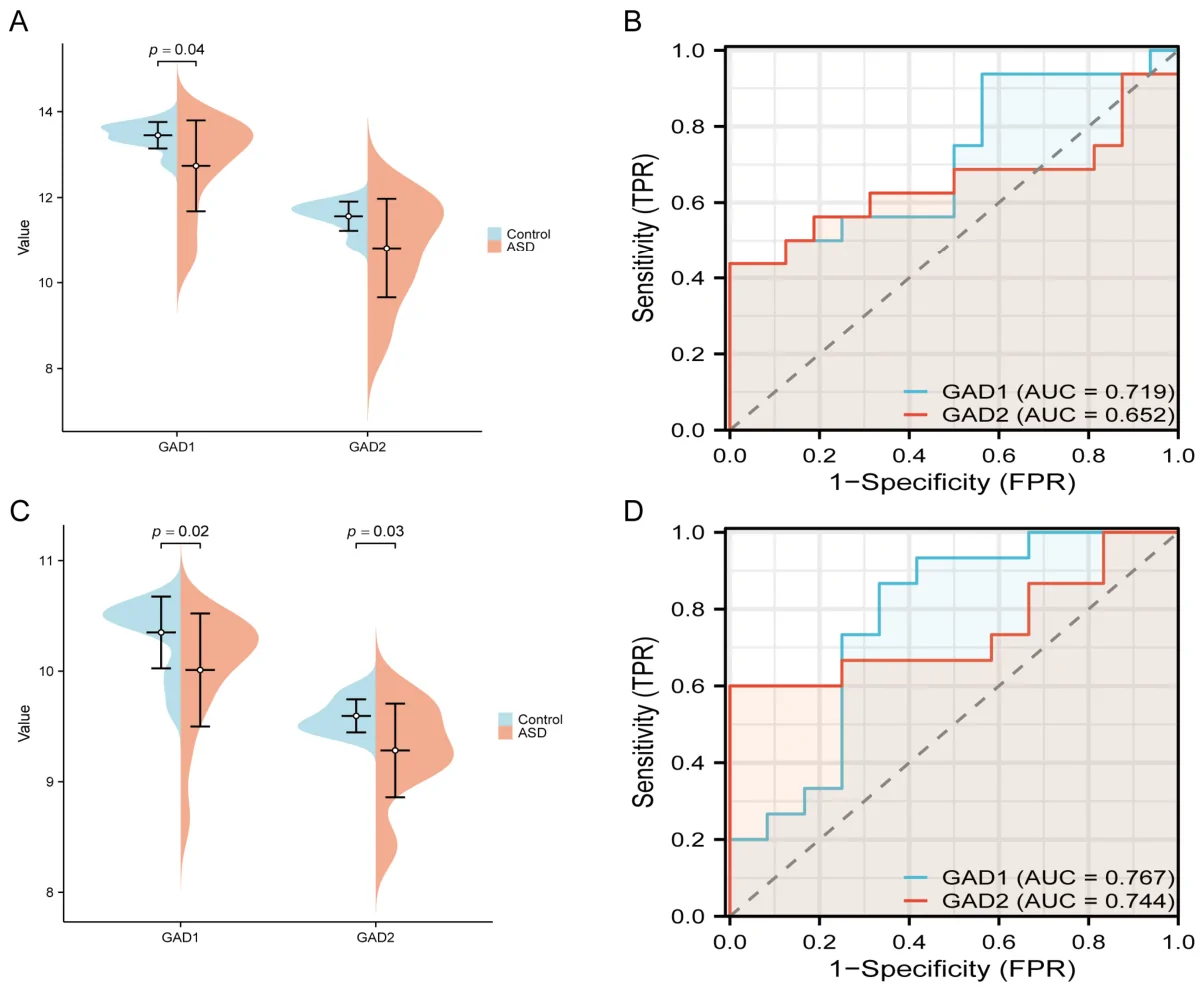
(**A**) Expression levels of hub gene targets in control and ASD groups within GSE28521 dataset. (**B**) ROC curve analysis of hub gene targets in GSE28521 dataset. (**C**) Expression levels of hub gene targets in control and ASD groups within GSE113834 dataset. (**D**) ROC curve analysis of hub gene targets in GSE113834 dataset.

**Figure 3:**
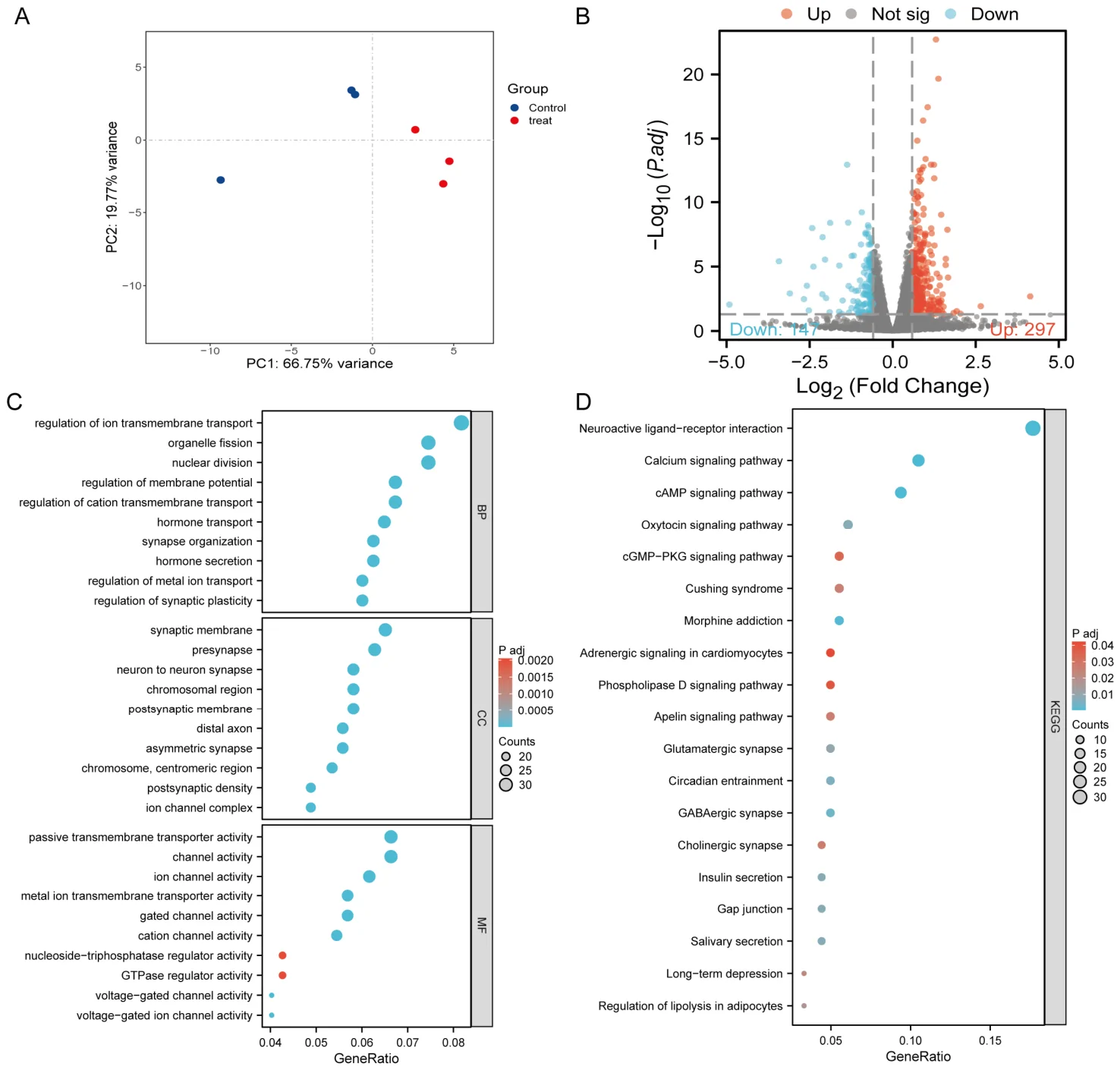
Transcriptomic analysis of E15 mouse forebrain samples exposed to TPHP. (**A**) Evaluation of data comparability. (**B**) Distribution of DEGs in volcano plot. (**C**) Bubble chart of GO enrichment analysis for DEGs. (**D**) Bubble chart of KEGG enrichment analysis for DEGs.

**Figure 4:**
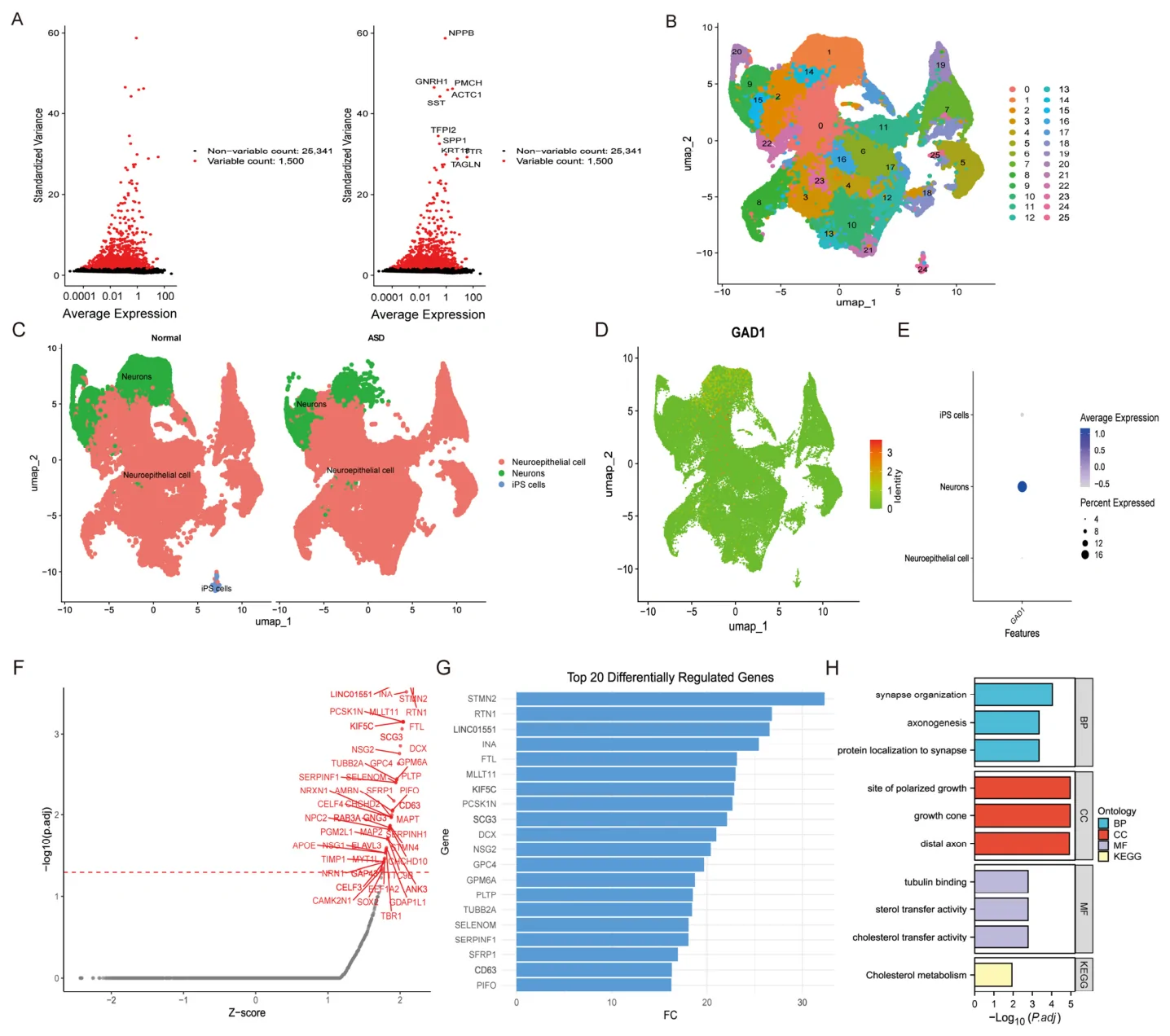
Single–cell transcriptomic analysis of core target genes. (**A**) Scatter plot of average gene expression versus normalized variance. (**B**) Uniform Manifold Approximation and Projection (UMAP) visualization showing clustered cells. (**C**) UMAP color–coded and annotated based on identified cell types. (**D**) UMAP plot highlighting spatial expression pattern of core target *GAD1* within cell populations. (**E**) Bubble chart depicting expression distribution of *GAD1* across cell subpopulations. (**F**) Volcano plot of significantly DEGs after *GAD1* knockout. (**G**) Bar chart of top 20 significantly DEGs after *GAD1* knockout. (**H**) Bar chart of GO and KEGG pathway analysis for significantly DEGs after *GAD1* knockout.

**Figure 5:**
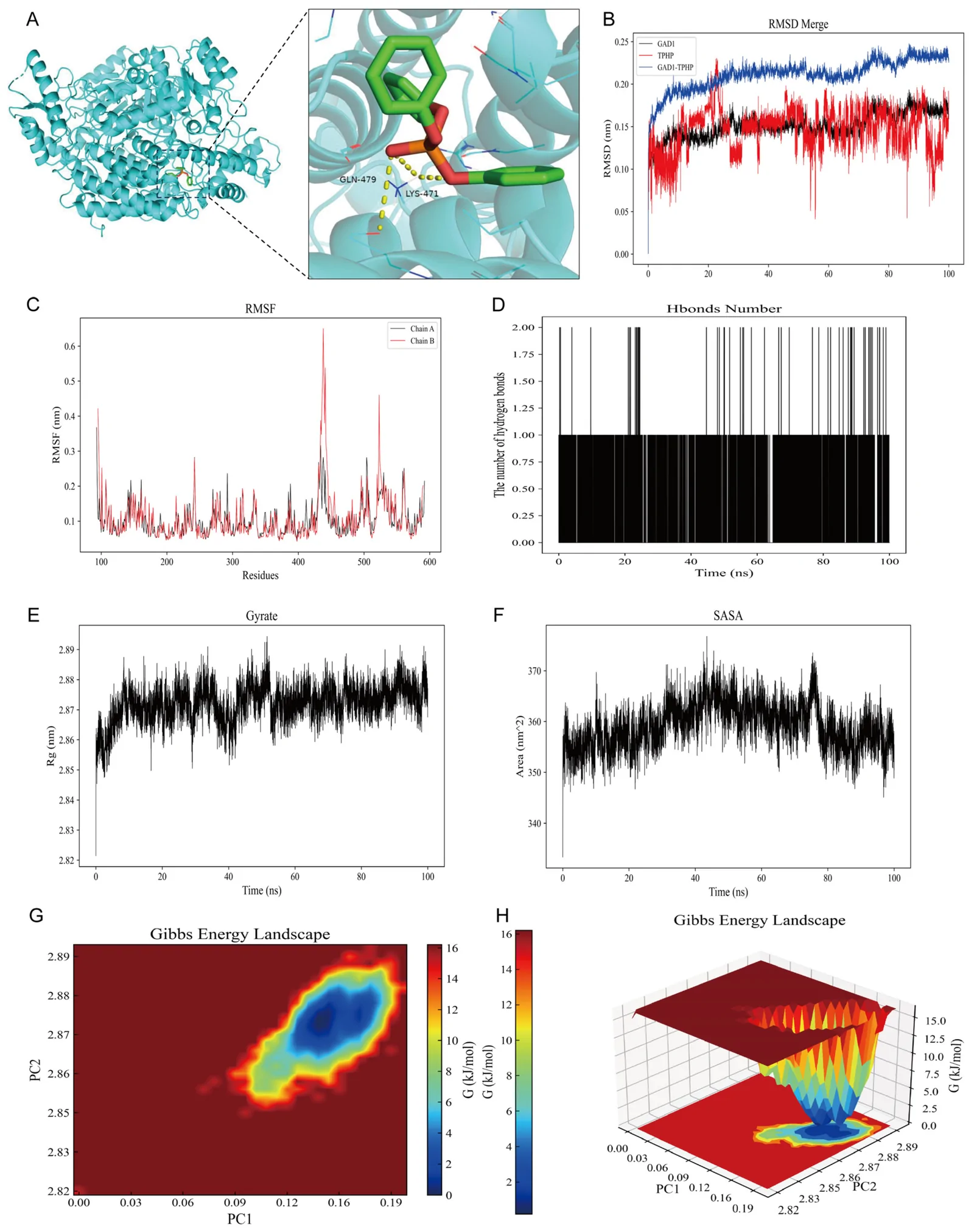
Molecular docking and molecular dynamic simulation. (**A**) Molecular docking shows binding affinity of TPHP to GAD1 protein. (**B**) Time–dependent root mean square deviation (RMSD) values of TPHP–GAD1 complex. (**C**) Root mean square fluctuation (RMSF) values of TPHP–GAD1 complex. (**D**) Number of hydrogen bonds in TPHP–GAD1 complex. (**E**) Radius of gyration (Rg) values of TPHP–GAD1 complex. (**F**) Solvent–accessible surface area (SASA) values of TPHP–GAD1 complex. (**G**,**H**) Gibbs free energy landscape of TPHP–GAD1 complex.

**Figure 6:**
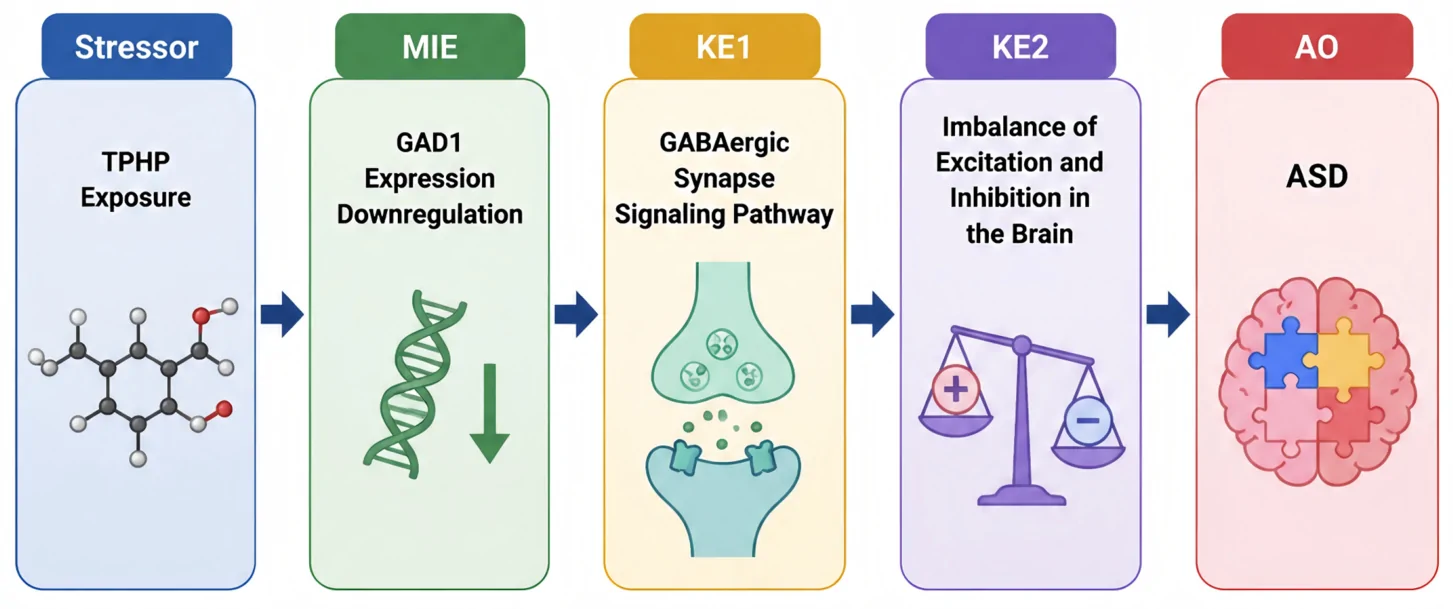
Proposed AOP model for ASD induced by TPHP exposure.

**Table 1:** Molecular mechanics/Poisson–Boltzmann surface area (MM–PBSA) analysis of TPHP–GAD1 complexes.

Contribution Components	TPHP–GAD1 (kcal/mol)
ΔVDWAALS	−41.20 ± 0.69
ΔEelec	−8.23 ± 0.28
ΔEGB	30.73 ± 0.94
ΔEsurf	−5.27 ± 0.03
ΔGgas	−49.43 ± 0.74
ΔGsolvation	25.45 ± 0.94
ΔTotal	−23.98 ± 1.20

## Data Availability

The data presented in this study are available on request from the corresponding author.

## Supplementary Materials

The following supporting information can be downloaded at https://www.mdpi.com/article/10.3390/ijms27198769/s1. References [55,56,102,103,104,105,106,107,108,109,110,111,112,113,114,115] are cited in Supplementary Materials.

## Abbreviations

The following abbreviations are used in this manuscript:

ASD	Autism spectrum disorder
TPHP	Triphenyl phosphate
OPFRs	Organophosphate flame retardants
DEGs	Differentially expressed genes
GO	Gene Ontology
KEGG	Kyoto Encyclopedia of Genes and Genomes
LASSO	Least absolute shrinkage and selection operator
RF	Random forest
SVM−RFE	Support vector machine recursive feature elimination
E15	Embryonic day 15
AOP	Adverse outcome pathway
