# Supplementary material for: Construction of Adverse Outcome Pathway (AOP) for Triphenyl Phosphate (TPHP)–Induced Autism Spectrum Disorder (ASD) and Risk Identification of Neurodevelopmental Toxicity of Aryl Organophosphorus Flame Retardants (OPFRs) Based on Multidimensional Bioinformatic Data
Source: Int J Mol Sci. 2026 Sep 30;27(19):8769. doi: 10.3390/ijms27198769 (PMC13664942; doi:10.3390/ijms27198769)

T4NPPP-GAD1:
-10.1kcal/mol

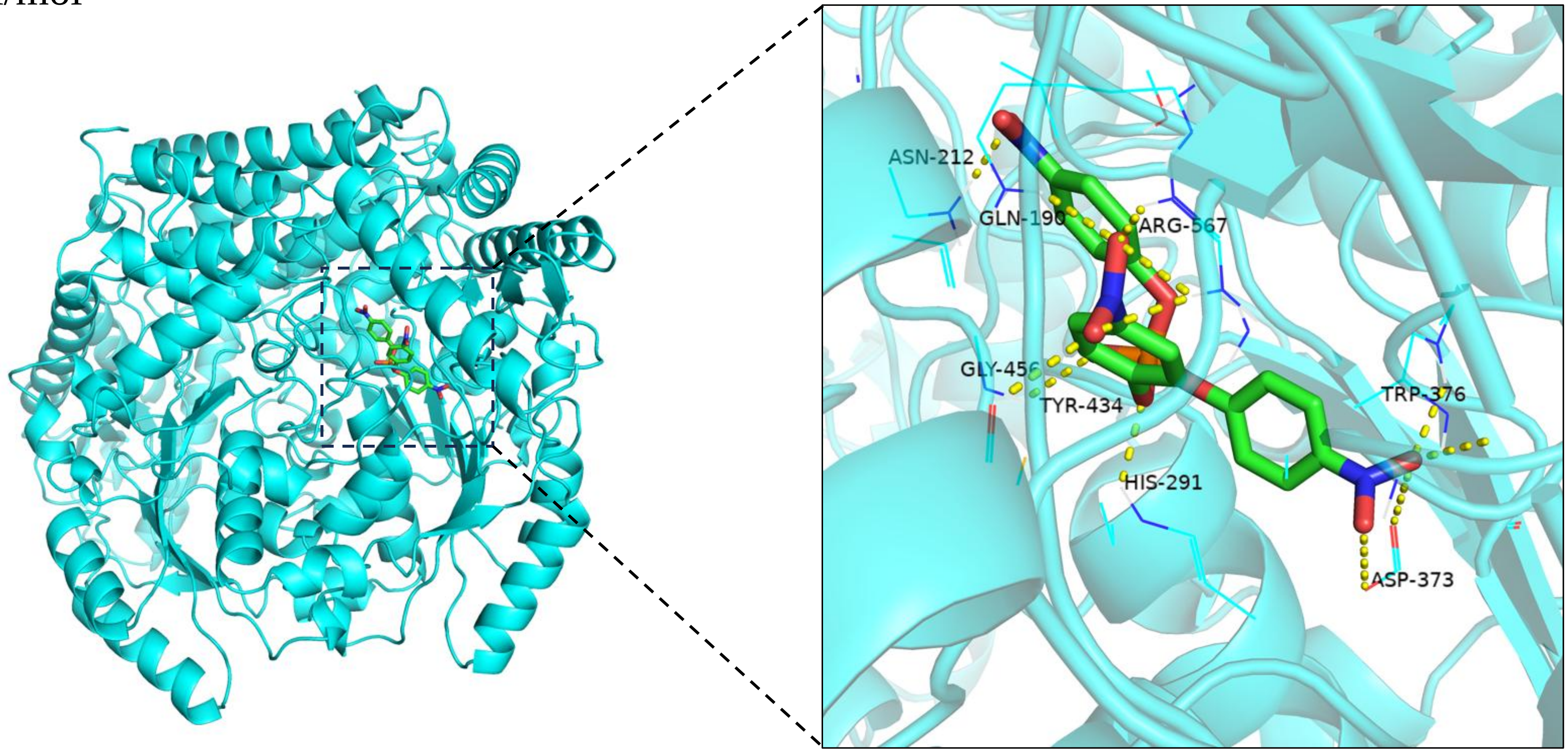

DP4PPP-GAD1:
-9.2kcal/mol

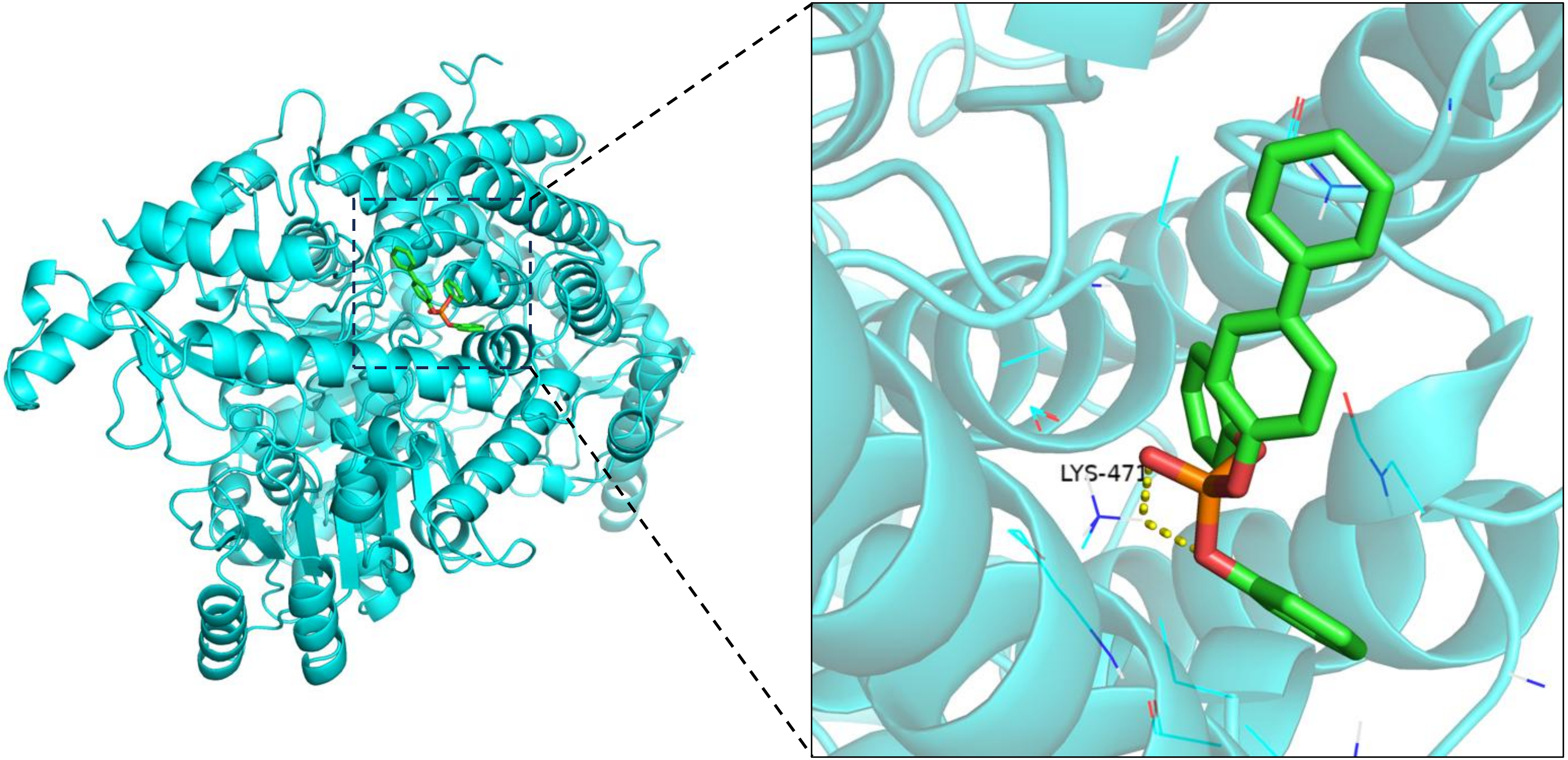

B24DTBPPTDP-GAD1:
-9.1kcal/mol

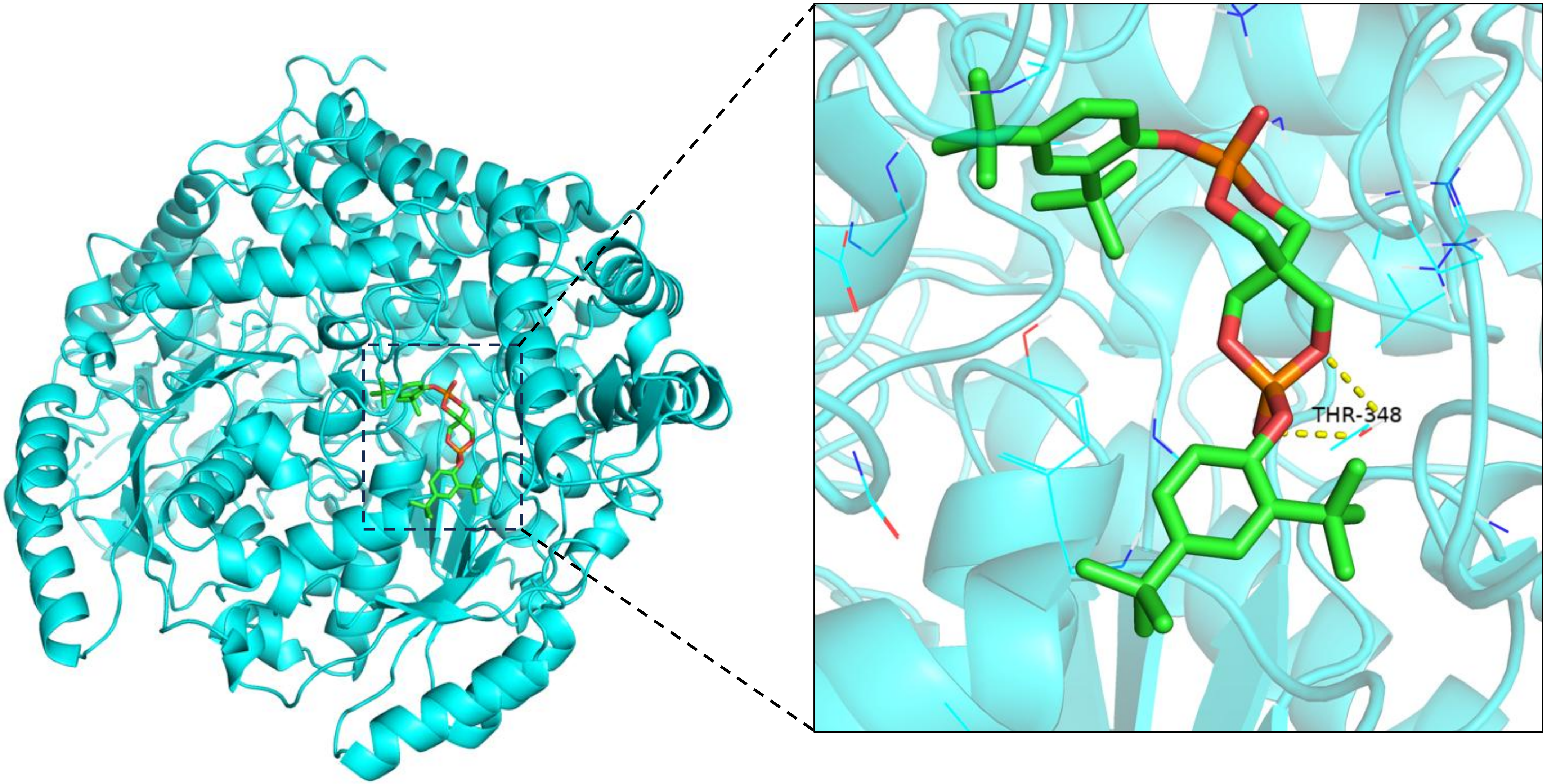

BDP-GAD1:
-8.9kcal/mol

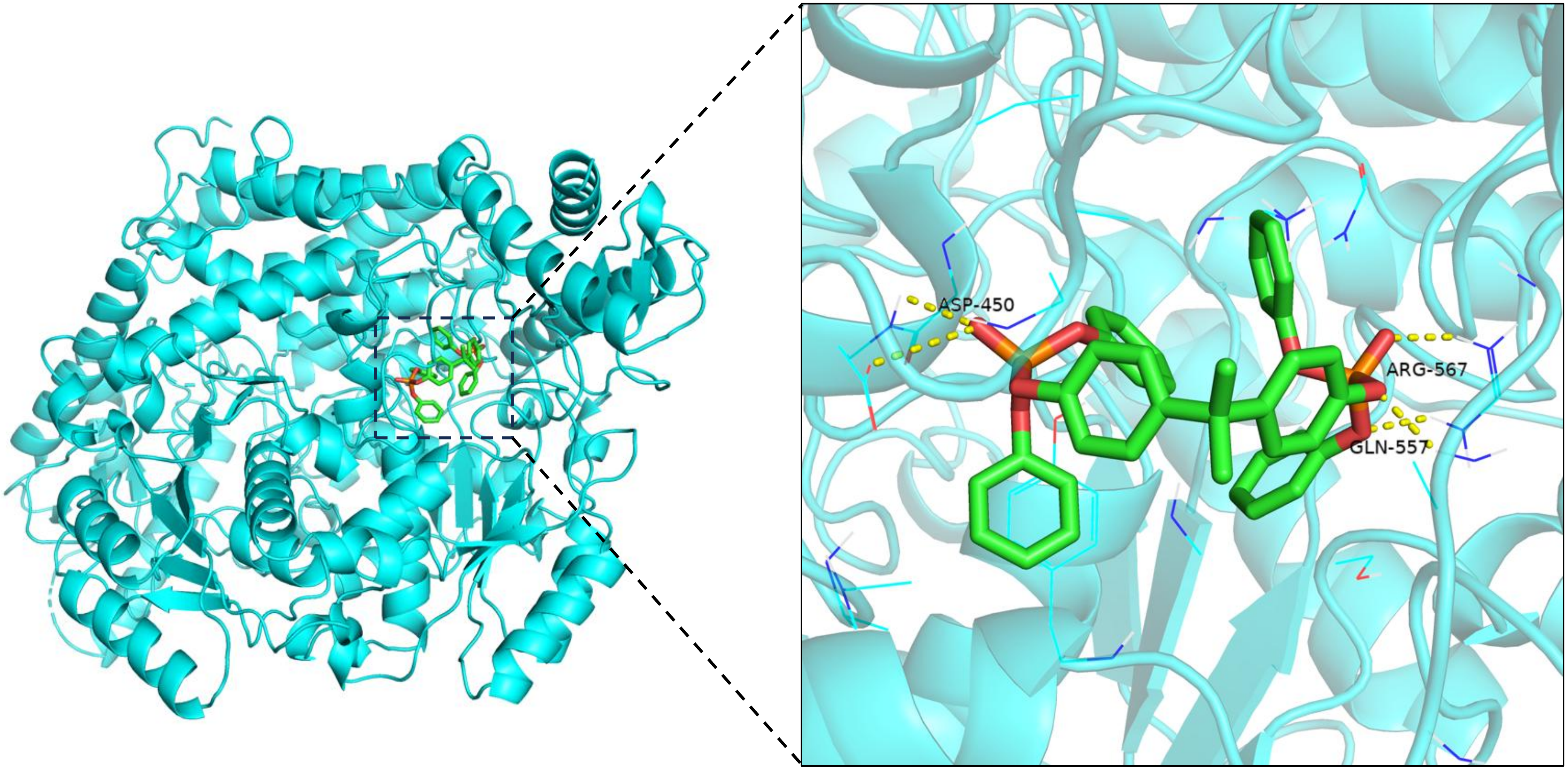

PBDMPP-GAD1:
-8.9kcal/mol

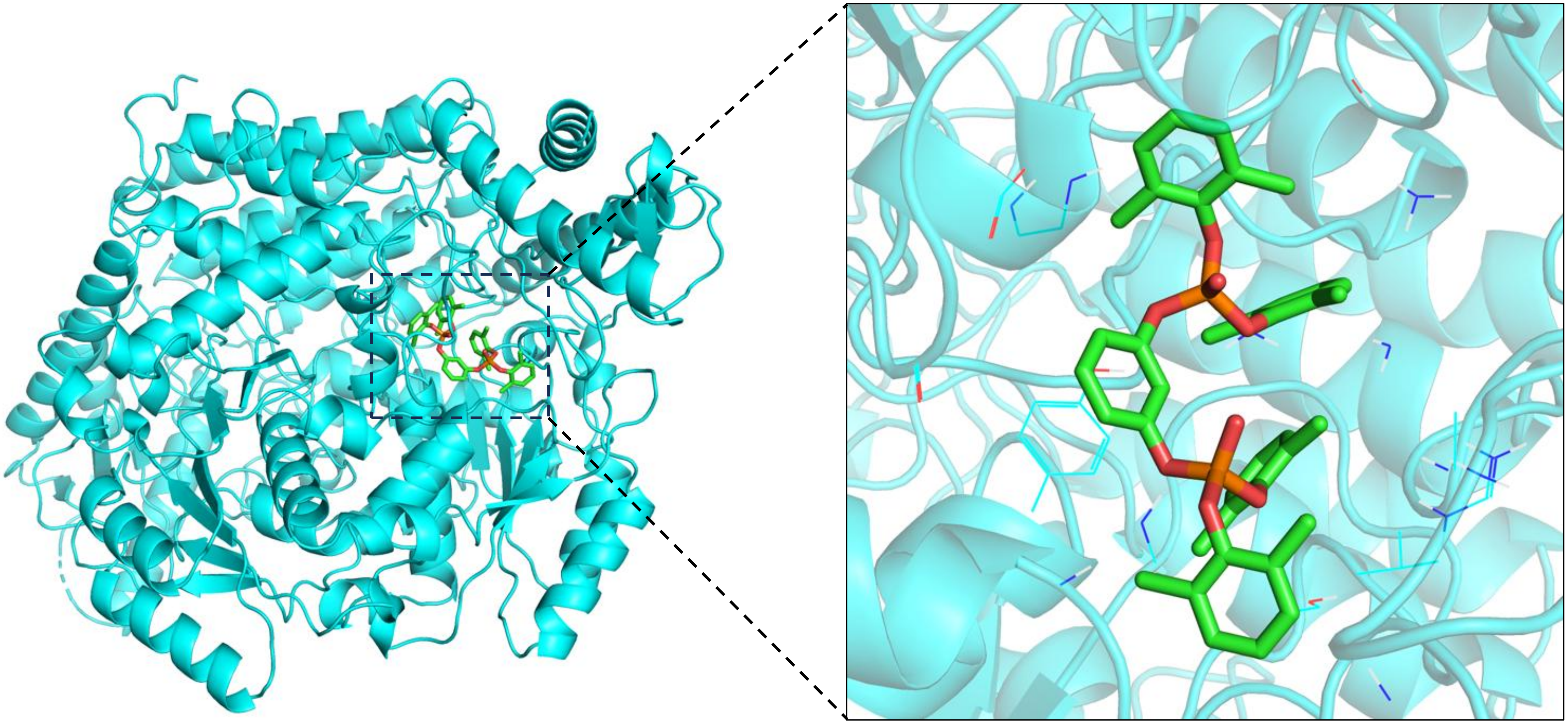

Supplement: Supplementary file 1 [file ijms-27-08769-s001.zip › Figures S1-S5.pdf]
