# Supplementary material for: Construction of Adverse Outcome Pathway (AOP) for Triphenyl Phosphate (TPHP)–Induced Autism Spectrum Disorder (ASD) and Risk Identification of Neurodevelopmental Toxicity of Aryl Organophosphorus Flame Retardants (OPFRs) Based on Multidimensional Bioinformatic Data
Source: Int J Mol Sci. 2026 Sep 30;27(19):8769. doi: 10.3390/ijms27198769 (PMC13664942; doi:10.3390/ijms27198769)
Supplement: Supplementary file 1 [file ijms-27-08769-s001.zip › Table S6.pdf]

Gene	OPFRs	Binding energy
GAD1	T4NPPP	-10.1
GAD1	DP4PPP	-9.2
GAD1	B24DTBPPTDP	-9.1
GAD1	BDP	-8.9
GAD1	PBDMPP	-8.9
GAD1	TIPPPs	-8.7
GAD1	B4IPPPP	-8.6
GAD1	2TBPDPP	-8.5
GAD1	3TBPDPP	-8.5
GAD1	3IPPDPP	-8.4
GAD1	IPPDPP	-8.4
GAD1	T3IPPP	-8.4
GAD1	T4IPPP	-8.4
GAD1	TDTBPP	-8.4
GAD1	T34DMPP	-8.1
GAD1	TXP	-8.1
GAD1	4IPPDPP	-8
GAD1	4-HDDP	-8
GAD1	B3IPPPP	-8
GAD1	B2TBPPP	-7.9
GAD1	B4TBPPP	-7.9
GAD1	TPHP	-7.9
GAD1	T4TBPP	-7.8
GAD1	TBPP	-7.8
GAD1	T246MPP	-7.7
GAD1	3OH-TPHP	-7.6
GAD1	4TBPDPP	-7.6
GAD1	T35DMPP	-7.6
GAD1	(E)-CHLORFENVINPH	-7.3
GAD1	CDP	-7.3
GAD1	TmMPP	-7.3
GAD1	B2IPPPP	-7.2
GAD1	BNDPP	-7.2
GAD1	EHDPP	-7.2
GAD1	T2MPP	-7.2
GAD1	T2iPPP	-7.1
GAD1	TMPP	-7.1
GAD1	Naftalofos	-7
GAD1	T26DMPP	-7
GAD1	24DIPPDPP	-6.9
GAD1	MDPP	-6.8
GAD1	2IPPDPP	-6.7
GAD1	BUDPP	-6.7
GAD1	DOPO	-6.7
GAD1	TEHP	-6.6
GAD1	ODPP	-6.4
GAD1	TIBP	-6.4
GAD1	Dimethylvinphos	-6.2
GAD1	(E)-Dimethylvinph	-6.1
GAD1	CEPPA	-6.1

GAD1	IDDP	-6.1
GAD1	TDCPP	-6.1
GAD1	TNPP	-6
GAD1	BEHPP	-5.9
GAD1	chlorpyrifos-oxon	-5.9
GAD1	DBPP	-5.9
GAD1	E-Mevinphos	-5.8
GAD1	(Z)-CHLORFENVINPH	-5.7
GAD1	DPPP	-5.7
GAD1	TIPP	-5.6
GAD1	Tetrachlorvinphos	-5.5
GAD1	PIP	-5.4
GAD1	Ragadan	-5.4
GAD1	TNBP	-5.4
GAD1	(Z)-DIMETHYLVINPH	-5.3
GAD1	D3HBP	-5.3
GAD1	DIDPP	-5.3
GAD1	DMPPP	-5.2
GAD1	MPP	-5.2
GAD1	(E)-Mevinphos	-5.1
GAD1	TPTP	-5.1
GAD1	(E)-Phosphamidon	-5
GAD1	THP	-4.9
GAD1	T13DCP2P	-4.7
GAD1	TAP	-4.7
GAD1	TPRP	-4.7
GAD1	Dibrom	-4.6
GAD1	FRC-6	-4.6
GAD1	T1CP2P	-4.6
GAD1	MP	-4.5
GAD1	T13DCPP	-4.5
GAD1	T23DCPP	-4.5
GAD1	TEP	-4.4
GAD1	TTBNPP	-4.4
