# Supplementary material for: Construction of Adverse Outcome Pathway (AOP) for Triphenyl Phosphate (TPHP)–Induced Autism Spectrum Disorder (ASD) and Risk Identification of Neurodevelopmental Toxicity of Aryl Organophosphorus Flame Retardants (OPFRs) Based on Multidimensional Bioinformatic Data
Source: Int J Mol Sci. 2026 Sep 30;27(19):8769. doi: 10.3390/ijms27198769 (PMC13664942; doi:10.3390/ijms27198769)
Supplement: Supplementary file 1 [file ijms-27-08769-s001.zip › Tables S4-S5.pdf]

Table S4. Assessment of the weight of evidence supporting essentiality of KEs

Events	Evidence	Weight of evidence
MIE		
Decreased GAD1	Direct evidence: GAD1 has been implicated in the pathophysiology of ASD and is downregulated in the frontal cortex of individuals with ASD[1-3]; Restoration of normal GAD1 expression can reduce neural abnormalities in the prefrontal cortex, thereby ameliorating ASD symptoms[4].	High
KE1		
Activation， GABAergic synapse signaling pathway	Direct evidence: Dysfunction of the GABAergic synapse signaling pathway leads to behavioral deficits in patients with ASD[5, 6]; Normal function of the GABAergic synapse signaling pathway contributes to the amelioration of ASD symptoms[4].	High
KE2		
Excitation-inhibition imbalance	Direct evidence: An imbalance in brain excitatory and inhibitory activity contributes to the pathogenesis and development of ASD[7-9]; Restoring the balance between excitatory and inhibitory signaling in the brain alleviates symptoms of ASD[10, 11].	High

Table S5. Assessment of the weight of evidence supporting KERs

Events	Evidence	Weight of evidence
MIE to KE1		
Downregulation of GAD1 leads to dysregulation of the GABAergic synapse signaling pathway	Biological plausibility[12];<br>Empirical support[13].	High
KE1 to KE2		
Dysfunction in GABAergic synaptic signaling disrupts the brain's excitatory-inhibitory (E/I) balance	Biological plausibility[5];<br>Empirical support[14, 15].	High
KE2 to AO		
E/I imbalance in the brain drives the initiation and progression of ASD	Biological plausibility[16];<br>Empirical support[7, 11].	High

[1] ZHUBI A, CHEN Y, GUIDOTTI A, et al. Epigenetic regulation of RELN and GAD1 in the frontal cortex (FC) of autism spectrum disorder (ASD) subjects [J]. Int J Dev Neurosci, 2017, 62: 63–72.

[2] PEARSON G, SONG C, HOHMANN S, et al. DNA Methylation Profiles of GAD1 in Human Cerebral Organoids of Autism Indicate Disrupted Epigenetic Regulation during Early Development [J]. Int J Mol Sci, 2022, 23(16).

[3] CHANG S C, PAULS D L, LANGE C, et al. Common genetic variation in the GAD1 gene and the entire family of DLX homeobox genes and autism spectrum disorders [J]. Am J Med Genet B Neuropsychiatr Genet, 2011, 156(2): 233–9.

[4] SINGLA R, MISHRA A, JOSHI R, et al. Homotaurine ameliorates the core ASD symptomatology in VPA rats through GABAergic signaling: Role of GAD67 [J]. Brain Res Bull, 2022, 190: 122–33.

[5] PIZZARELLI R, CHERUBINI E. Alterations of GABAergic signaling in autism spectrum disorders [J]. Neural Plast, 2011, 2011: 297153.

[6] GINIATULLIN R, CHERUBINI E. Gabaergic signalling in Autism Spectrum disorders (ASD): Role of glial cells and therapeutic perspectives [J]. Brain Behav Immun, 2025, 129: 681–9.

[7] SHIN D, CHO E, PARK K, et al. Early postnatal exposure to bicuculline modulates E/I balance and induces ASD-like behavioral phenotypes in mice [J]. Anim Cells Syst (Seoul), 2025, 29(1): 264–81.

[8] CULOTTA L, PENZES P. Exploring the mechanisms underlying excitation/inhibition imbalance in human iPSC-derived models of ASD [J]. Mol Autism, 2020, 11(1): 32.

[9] BOZZI Y, PROVENZANO G, CASAROSA S. Neurobiological bases of autism-epilepsy comorbidity: a focus on excitation/inhibition imbalance [J]. Eur J Neurosci, 2018, 47(6): 534–48.

[10] ZHANG J, EATON M, CHEN X, et al. Restoration of excitation/inhibition balance enhances neuronal signal-to-noise ratio and rescues social deficits in autism-associated Scn2a-deficiency [J]. bioRxiv, 2025.

[11] COLOMAR L, SAN JOSé CáCERES A, ÁLVAREZ-LINERA J, et al. Role of cortical excitatory/inhibitory imbalance in autism spectrum disorders from a symptom severity trajectories framework: a study protocol [J]. BMC Psychiatry, 2023, 23(1): 213.

[12] BENNER O, KARR C H, BARTOL T M, et al. Presynaptic Trafficking of Glutamate Decarboxylase Isoforms Is Dispensable for Basal GABAergic Neurotransmission [J]. J Neurosci, 2026, 46(2).

[13] CHATTOPADHYAYA B, DI CRISTO G, WU C Z, et al. GAD67-mediated GABA synthesis and signaling regulate inhibitory synaptic innervation in the visual cortex [J]. Neuron, 2007, 54(6): 889–903.

[14] FLORES-BARRERA E, THOMASES D R, TSENG K Y. MK-801 Exposure during Adolescence Elicits Enduring Disruption of Prefrontal E-I Balance and Its Control of Fear Extinction Behavior [J]. J Neurosci, 2020, 40(25): 4881–7.

[15] CHEN X, PAN D, LIU J J, et al. Endophilin A1 facilitates organization of the GABAergic postsynaptic machinery to maintain excitation-inhibition balance [J]. Elife, 2025, 13.

[16] UZUNOVA G, PALLANTI S, HOLLANDER E. Excitatory/inhibitory imbalance in autism spectrum disorders: Implications for interventions and therapeutics [J]. World J Biol Psychiatry, 2016, 17(3): 174–86.
